# Relative contributions of CA3 and medial entorhinal cortex to memory in rats

**DOI:** 10.3389/fnbeh.2014.00292

**Published:** 2014-08-28

**Authors:** Kally C. O'Reilly, Juan M. Alarcon, Janina Ferbinteanu

**Affiliations:** ^1^Center for Neural Science, New York UniversityNew York, NY, USA; ^2^Department of Pathology, The Robert F. Furchgott Center for Neural and Behavioral Science, SUNY Downstate Medical CenterBrooklyn, NY, USA; ^3^Division of Neuroscience, Department of Physiology and Pharmacology, SUNY Downstate Medical CenterBrooklyn, NY, USA

**Keywords:** CA3, medial entorhinal cortex, spatial memory, Schaffer collaterals, temporo-ammonic pathway

## Abstract

The hippocampal CA1 field processes spatial information, but the relative importance of intra- vs. extra-hippocampal sources of input into CA1 for spatial behavior is unclear. To characterize the relative roles of these two sources of input, originating in the hippocampal field CA3 and in the medial entorhinal cortex (MEC), we studied effects of discrete neurotoxic lesions of CA3 or MEC on concurrent spatial and nonspatial navigation tasks, and on synaptic transmission in afferents to CA1. Lesions in CA3 or MEC regions that abolished CA3-CA1, or reduced MEC-CA1 synaptic transmission, respectively, impaired spatial navigation and unexpectedly interfered with cue response, suggesting that in certain conditions of training regimen, hippocampal activity may influence behavior otherwise supported by nonhippocampal neural networks. MEC lesions had milder and temporary behavioral effects, but also markedly amplified transmission in the CA3-CA1 pathway. Extensive behavioral training had a similar, but more modest effect on CA3-CA1 transmission. Thus, cortical input to the hippocampus modulates CA1 activity both directly and indirectly, through heterosynaptic interaction, to control information flow in the hippocampal loop. Following damage to hippocampal cortical input, the functional coupling of separate intra- and extra-hippocampal inputs to CA1 involved in normal learning may initiate processes that support recovery of behavioral function. Such a process may explain how CA3 lesions, which do not significantly modify the basic features of CA1 neural activity, nonetheless impair spatial recall, whereas lesions of EC input to CA1, which reduce the spatial selectivity of CA1 firing in foraging rats, have only mild effects on spatial navigation.

## Introduction

Multiple studies in rats have shown that the integrity of hippocampal CA1 field activity is necessary for the encoding and long-term storage of spatial information (Auer et al., 1989; Remondes and Schuman, 2004; Lee et al., 2005; Poirier et al., 2008; Song et al., 2009). Recent work has focused on the contributions of CA1 inputs to generating the characteristics of place-selective activity in CA1. Consistent with their putative role in memory, CA1 neurons receive no direct sensory information, but rather highly processed multimodal input from the entorhinal cortex (EC), which sends information directly, through the temporoammonic pathway, and indirectly, through the dentate gyrus (DG)-CA3-CA1 loop (the trisynaptic circuit; Amaral and Witter, 1995; Witter and Amaral, 2004). CA3 lesions do not significantly modify CA1 activity features despite associated impairment in spatial recall (Brun et al., 2002). In contrast, lesions of EC input to CA1 reduce the spatial selectivity of CA1 firing in foraging rats (Brun et al., 2008). However, understanding the importance of these results in the context of behavioral spatial function is hampered by the distinct memory requirements of the behavioral tasks, which may modulate the properties of CA1 activity (Smith and Mizumori, 2006; Ferbinteanu et al., 2011). Data from other studies further contribute to ambiguity, as EC lesions produce behavioral deficits in some (Schenk and Morris, 1985; Nagahara et al., 1995; Good and Honey, 1997; Oswald and Good, 2000; Oswald et al., 2003), but not all cases (Pouzet et al., 1999; Bannerman et al., 2001; Burwell et al., 2004; Parron et al., 2006). Similarly, effects of CA3 neurotoxic lesions, or other experimentally-induced dysfunctions of CA3, have been interpreted as either impairing spatial memory (Nakazawa et al., 2003; Lee and Kesner, 2004) or object-place associations (Hunsaker and Kesner, 2008; Langston et al., 2010), leaving the issue of CA3 function in spatial memory unresolved (Kesner, 2007; Gilbert and Brushfield, 2009; Langston et al., 2010). To our knowledge, no recent experiment directly compared the relative contributions of EC and CA3 to memory-based performance.

**Figure 1:**
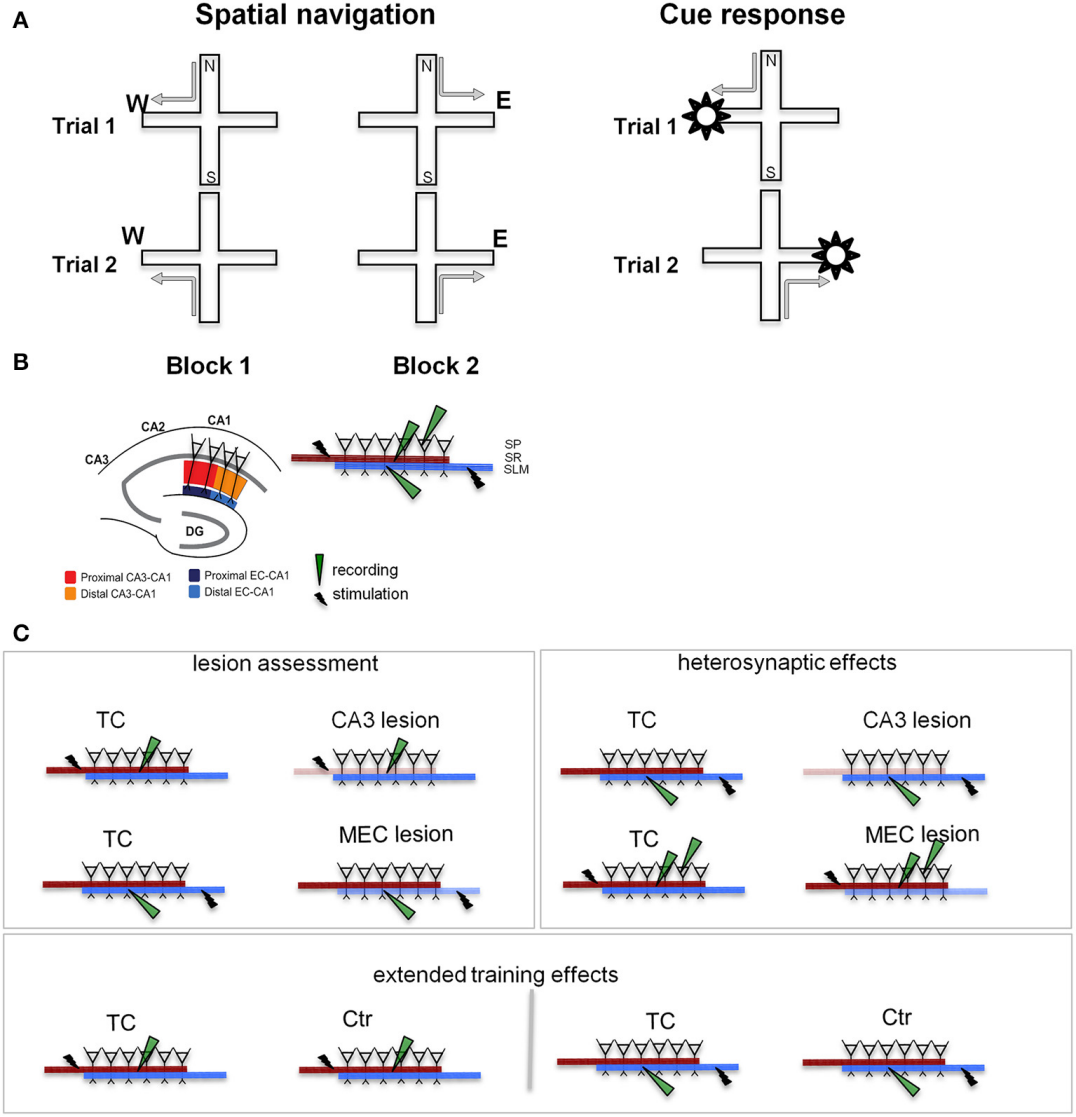
**Behavioral training and testing were followed by slice recording. (A)** Experimental design. We TC animals in two different tasks on the plus maze: a spatial navigation task (left) and a cue-response task (right). In both cases, the rats walked from one of a pair of opposite start arms to the goal arm that was baited on the particular trial. In the spatial memory task the animals had to remember the location of the food, while in the cue response task they had to remember a cue-motor response association. Thus, the rat had to choose and consistently use the appropriate strategy. Neither task involved spatial or body turn alternation (see Supplementary Figure 1), but required either spatial navigation, or a cue-response strategy. **(B)** Stimulation and recording in hippocampal slices. The segregation of inputs to CA1 field permits evaluation of CA3 and MEC lesions on the neurophysiology of the local CA1 circuit. Separate sets of recordings were performed for the proximal (close to CA3) and distal (close to subiculum) areas of the CA1. The inset details the placement of stimulation/recording electrodes in stratum radiatum (SR), stratum lacunosum-moleculare (SLM), and stratum pyramidale (SP), where the somas of CA1 neurons are located. Synaptic transmission in the CA3-CA1 pathway was assessed by stimulating and recording fEPSPs in the stratum radiatum (red and orange). Synaptic transmission in the EC-CA1 pathway was assessed by stimulating and recording fEPSPs in the stratum lacunosum-moleculare (navy and blue). CA1 neural firing in response to stimulation of the CA3 input was assessed by stimulating in the stratum radiatum and recording in the stratum pyramidale. Evaluation of CA3-CA1 and EC-CA1 synaptic transmission was performed in four groups: Ctr, TC, CA3 lesion, and MEC lesion. Ctr group received minimal behavioral training, while the TC and lesion groups had extensive behavioral experience (for details, see *Methods*). DG, dentate gyrus; CA3-CA1, cornu ammonis 3-cornu ammonis 1. **(C)** Electrophysiological recording experiments. CA3 and MEC lesion effects (left panel) were assessed by evaluating synaptic transmission in the SR (top row) and SLM (bottom row), respectively. Heterosynaptic lesion effects (right panel) were assessed by measuring synaptic transmission in the SLM in animals with CA3 lesions (top row), and synaptic transmission in the SR together with neural population firing in SP in animals with MEC lesions (bottom row). Behavioral training effects (bottom) were evaluated by comparing synaptic transmission SR (left) and SLM (right) in TC and Ctr groups. Lighter color indicates the damaged pathway in lesioned groups.

The goal of the present study was to characterize the roles of EC and CA3 in the retention of a behavioral paradigm involving the concurrent acquisition of two memory-based strategies, spatial navigation and response to a visible cue (Figure 1A, Supplementary Figure 1). Because MEC is primarily involved in processing spatial information (McNaughton et al., 1983; Burwell, 2000; Hafting et al., 2005; Hargreaves et al., 2005; Knierim et al., 2014), our study focused on this area, rather than the entire EC. The behavioral paradigm was initially designed by Tolman and colleagues to study place vs. response learning (Tolman et al., 1946). In the original version, rats were trained to run from the stem of a T-shaped maze in one of the two side arms to obtain a food pellet. The maze was then rotated 180° and the rats could continue to go to the old location by making a different body turn (a cognitive map strategy), or maintain the body turn and reach the opposite location (a stimulus-response type of strategy). Subsequent extensive studies demonstrated distinct contributions of the hippocampal and dorsal striatum, respectively, to the two memory-dependent strategies (Packard et al., 1989; Packard and White, 1991; Packard and McGaugh, 1992, 1996; McDonald and White, 1994). We modified this paradigm to study hippocampal activity by using a plus-maze and recording CA1 unit activity in rats trained to find food in one of a pair of opposite goal arms when coming from either start arm. The location of the food was changed regularly during the session and to behave efficiently, the rat had to permanently monitor the food location relative to his own. Our unit recordings showed that when rats perform in this paradigm, the CA1 activity forms a temporally organized representation of the environment (Ferbinteanu and Shapiro, 2003), which presumably supports the rats' ability to navigate efficiently. A different, nonspatial variant required the animals to find food by following a visible cue moved randomly between the two goal arms. In this version, the allocentric cues remain present, but they do not have informative value for reaching the goal, which is signaled by the intramaze visible cue. The efficient behavioral strategy in this case is to ignore spatial information and walk toward the cue regardless (stimulus-response). Hippocampal dysfunction rendered the rats unable to make the “correct” choice based on spatial location, while, as expected, it had no effect if the rats were trained to follow the visible cue (Ferbinteanu and Shapiro, 2003; Ferbinteanu et al., 2011). These data support the idea that the hippocampal activity is necessary for spatial navigation, but is not involved in storing a cue-response association or in attention, motivation, motor ability, or memory for the basic rules of the tasks. Here, we trained rats concurrently in both the spatial and nonspatial versions of the task to test the effects of CA3 and MEC neurotoxic lesions (Supplementary Figure 1). We predicted that both types of lesion would affect spatial navigation but leave cue-response behavior intact. We could not predict *a priori* which lesion would have more severe effects, particularly as previous literature suggests inconsistent effects of EC damage on spatial navigation (see also Aggleton et al., 2000).

To characterize the physiological changes of the CA1 local circuits associated with behavioral modifications following MEC and CA3 lesions, we followed behavioral testing with *in vitro* physiological recording of the synaptic response of CA1 neurons (Figure 1B). The CA3 and MEC pathways to CA1 are well segregated and topographically organized (Amaral and Witter, 1995; Witter and Amaral, 2004). CA3 neurons contact the dendrites of CA1 neurons through the Schaffer collaterals close to CA1 pyramidal soma, in the stratum radiatum, while the EC neurons send input to CA1 through the temporo-ammonic pathway at the distal tip of the apical dendrites, in the stratum lacunosum-moleculare. MEC projects predominantly to the proximal (close to DG) CA1, while lateral EC (LEC) projects predominantly to the distal (close to subiculum) CA1. We therefore predicted that we would find marked effects in the stratum radiatum after CA3 lesions and in the proximal stratum lacunosum-moleculare after MEC lesions. We assessed synaptic function in the lesioned pathways (Figure 1C, left), heterosynaptic interactions between MEC-CA1 and CA3-CA1 pathways (Figure 1C, right), and effects of behavioral training (Figure 1C, bottom).

## Materials and methods

### Subjects

Male Long-Evans rats (300–350 g, 4–6 months old, Charles River Labs) were housed in individual cages (12 h light cycle) and food deprived to 85-90% of body weight before and during behavioral training. All procedures with animals met NIH guidelines and were approved by the SUNY Downstate Medical Center Institutional Animal Care and Use Committee. Procedures were designed to minimize the numbers of subjects used, involving extensive preoperative training to allow comparison of individual subject data relative to each rat's preoperative baseline.

### Apparatus

The plus maze was made of gray polyvinyl chloride (PVC) and elevated 91 cm from the floor of a room that contained several visual cues. Each of 4 arms was 61 cm long and 6.3 cm wide. A gray PVC block (30.4 cm high, 6.3 cm wide, 15.2 cm deep) was used to block the start arm that was not in use for that trial. Each goal arm had at its distal end a sunk well covered with a wire mesh. Each well contained a piece of fruit loop throughout testing; thus, the baited and the empty goal arms did not differ in odor. A rectangular waiting platform (32 × 42 cm) was placed next to the maze. In the cued version of the task, a white visible flag made of PVC was used to indicate the location of the food on the maze.

### Behavioral training and testing

Two tasks were employed for this study: a spatial navigation task and a cue-response task. Training procedures followed previously established protocols (Ferbinteanu and Shapiro, 2003; Ferbinteanu et al., 2011; Supplementary Figure 1). Behavioral training, lesion, physiology, and histology procedures for each animal are presented in Supplementary Table 1. Most animals were trained on both tasks (see below), starting with the cue-response and adding the spatial navigation when the basic structure of the task was acquired (i.e., rats walked quickly from the start position to retrieve food in one of the goal arms). Thus, the acquisition of the two types of behavioral strategies occurred concurrently. All animals were pre-exposed to the maze in the presence of food for 2 consecutive days and then trained to walk from either the North or the South start arms to the end of West or East goal arms to obtain half a Fruit Loop. Between trials, the rats were placed on a side platform to wait for the next trial. Entry with all four paws into the unrewarded arm defined an *error*, which the rat was allowed to correct. When the animals were trained in the two tasks (see below), the order of presentation within a session was changed daily. The start and the goal arm were selected based on a pseudorandom sequence of 60 trials with ≤3 consecutive repetitions of the same type of journey (NE, NW, SE, or SW). In the *spatial task*, the location of the food was kept constant for a maximum of 20 trials (15 trials post-surgery), or until the rat entered the correct goal arm in 9 of 10 consecutive trials. At that point, the other goal arm was baited and a new block of trials began. Alternating blocks continued up to either 4 blocks or 60 total trials. In the *cue task*, the rat had to walk toward a visible cue (a white L-shaped object), whose position, like the start position, was changed pseudorandomly between the goal arms and on which the food was placed. Training continued until the rat reached a criterion of 80% correct trials on 2 consecutive days either, (a) in the single task it was trained on or (b) in both tasks, when it was trained in both the spatial and cue-response paradigms. Two sets of animals were used.

#### Set1: control for physiological procedures

This set was constituted of three subgroups: (1a) cage controls (*n* = 2), which were handled, placed on food restriction, and brought to the lab, but were not subjected to experimental training; (1b) animals trained in the spatial strategy until reaching 80% correct performance for 2 consecutive days (*n* = 2); (1c) animals trained in the cue strategy until reaching 80% correct performance for 2 consecutive days (*n* = 2). The purpose of these groups was to provide a control for investigating the effect of extensive training on the functionality of the local CA1 circuitry, and physiological recordings from these animals were combined in one control (Ctr) group.

#### Set2: experimental groups for characterizing CA3 and MEC involvement using CA3 or MEC specific lesions

This set was also constituted of 3 subgroups: (2a) trained control (TC); (2b) CA3 lesion; (2c) MEC lesion. All animals were handled, placed on food restriction, and trained to 80% correct criterion on *both* the cue response and spatial navigation tasks, after which they were randomly assigned to one of three subgroups and underwent surgical procedures (2a: sham, who were anesthetized and received an incision of the scalp, which was subsequently sutured; 2b: NMDA neurotoxic lesion of CA3; 2c: NMDA neurotoxic lesion of MEC). Following a recovery period of approximately 1 week, they were subsequently re-tested for either 2 days (*n* = 4 MEC lesion) or 5 consecutive days (*n* = 5 CA3 lesion; *n* = 2 MEC lesion; *n* = 4 TC).

Post- lesion, the size of the trial block in the spatial task was set at maximum of 15 trials so that the goal arms were reinforced in a balanced manner. If the rat did not reach the 9/10 correct criterion in 15 successive trials, the location of the food was shifted to the other goal arm and the corresponding block of trials was denoted as a *long block* (Supplementary Figure 1). At the end of behavioral procedures, the animals were overdosed with isoflurane and the brain tissue processed for slice physiology and/or for histological assessment of the lesion.

### Behavioral data analysis

For final analysis, we included only data from animals with selective and extensive lesions in the targeted areas (see last column in Supplementary Table 1). Percent performance error was calculated for each rat during each day of testing and a mean was calculated for each subgroup during each day. We also computed the proportion of *long blocks* for each animal during each spatial navigation test. Differences in performance were assessed by using a mixed models analysis. Because the missing values in the study may be dependent on the observed outcomes (that is, post-lesion testing for four of the six MEC was terminated when they reached 80% correct criterion) we assumed that that the missing value mechanism to be missing at random (MAR) rather than missing completely at random (MCAR). Hence, a mixed models approach, which gives unbiased estimates under MAR, was considered to be the best choice for the longitudinal analysis. This approach takes into account the within-subject correlation of the repeated measurements. Time (in days) as a categorical independent variable, and performance (as percent error on repeated time points) as a dependent variable were entered into the mixed model. Unstructured covariance was the primary candidate for fitting the covariance structure in mixed models analysis. Other covariance structures such as Autoregressive (AR), Compound Symmetry (CS), and Variance Components (VC) were also considered and the best structure was chosen using model selection strategies such as Akaike's Information Criteria (AIC), Bayesian Information Criteria (BIC) and plots of residual correlations and lag covariances. The means reported in **Figure 5** are the least squares means (marginal means). The degrees of freedom were computed according to Sattherthwaite formula, which takes into consideration the variance within the group along with the sample size, and is robust against heterogeneity of variance. All analyses were done using SAS version 9.2 (SAS Institute, Inc., NY).

#### Post-hoc power analysis for spatial navigation results

In order to check whether the sample sizes were sufficient to detect (a) overall main effect between CA3 and controls, (b) overall main effect between MEC and controls, and (c) difference between the lesions at day 5, post-lesion, we conducted a *post-hoc* power analysis at 5% significance level based on the formula:

(1) $$Power=\Phi \left\{-Z_{1-\frac{\alpha }{2}}+\frac{\Delta }{\sqrt{\frac{\sigma_{1}^{2}}{n_{1}}+\frac{\sigma_{2}^{2}}{n_{2}}}}\right\},$$

where Φ = tail area under the standard normal density function,

n_1_ = sample size for group 1,

n_2_ = sample size for group 2,

Z = critical Z value for a given α,

α = probability of type I error (0.05),

σ_1_, σ_2_ = variance of mean for group 1 and mean for group 2,

Δ = |μ_2_ − μ_1_|= absolute difference between the means of groups 1 and 2.

We did not consider power for the overall main effect between lesion groups since our hypothesis, based on previous literature (Morris et al., 1982; Ferbinteanu et al., 1999; Burwell et al., 2004; Parron et al., 2004, 2006), was that both lesions would impair spatial navigation. Rather, we were interested in the question of whether the two types of lesions affected performance differently as animals underwent training post-surgery. We accompanied power analysis by calculating the effect sizes:

(2) $$ES=\Delta /\sigma_{p,}$$

where Δ is as defined above and σ_*p*_is the pooled standard deviation (Cohen, 1988).

#### Post-hoc power analysis for cue response results

Previous literature (e.g., McDonald and White, 1994; Packard and McGaugh, 1996), suggested that neither lesion would have effects on cue responses. Although we found a similar pattern, but smaller effect sizes for cue responses as for spatial navigation, we did not have an a priori hypothesis. Hence we consider the results of the cue-response task as exploratory.

### Lesions

Rats were anesthetized with isoflurane and diazepam or midazolam (10 mg/kg). Atropine (5 mg/kg body weight) was also administered in order to avoid fluid accumulation in the respiratory tract. Neurotoxic lesions were made by injecting a solution of 6 mg/ml NMDA in phosphate buffer (pH 7.4) through a 30-gauge cannula attached to a minipump (0.2 μl/min; New Era Pump Systems, Inc., Model NE-4000). At the end of each injection, the cannula was left in place for 3 min, retracted 0.5 mm and left in this location for 1 min, and then slowly retracted completely. The coordinates of each injection and the volumes injected are presented in Table 1. In order to prevent seizure development, a second, ip., injection of valium (10 mg/kg body weight) was administered prior to neurotoxin infusion and animals were monitored until completely awake and active in their home cages. Sham animals were anesthetized, incised and sutured.

**Table 1:** **Coordinates of CA3 and MEC lesions**.

**CA3**	**MEC**
1. AP-2.8; L ± 2.6; V-3.7 0.25 μl	1. AP-7.8; L ± 4.5; V-6.8 0.25 μl
2. AP-3.3; L ± 3.0; V-3.8 0.25 μl	2. AP-8.3; L ± 4.5; V-5.8 0.25 μl
3. AP-4.1; L ± 3.4; V-4.0 0.25 μl	3. AP-8.8; L ± 4.5; V-4.8 0.25 μl
4. AP-4.1; L ± 4.2; V-4.4 0.25 μl	
5. AP-4.8; L ± 4.1; V-4.4 0.25 μl	
6. AP-4.8; L ± 4.6; V-6.7 0.25 μl	
7. AP-5.6; L ± 4.4; V-5.3 0.25 μl	

AP, antero-posterior from bregma; L, lateral from bregma; V, ventral from bregma. All coordinates are in mm. Coordinates were determined based on previous work (e.g., Ferbinteanu et al., 2003) and pilot work for this study.

### Retrograde tracer

To verify the extent of the MEC lesions using a method other than cresyl violet histological staining, we used retrograde tracing in a separate group of three MEC lesioned and two control animals. The controls received bilateral FluoroGold (FG) injections (0.35 μl/injection of 2% solution) in the dorsal CA1 (1: AP -3.8 mm; ML: ± 2.6 mm; DV: -2.8 mm; 2: AP -5.2 mm; ML: ±4.2 mm; DV: -3.2 mm). Rats with MEC lesions received bilateral FG injections into dorsal CA1 1 week after the MEC lesions were performed. The interval between the lesion and FG injections was similar to the interval between lesion and behavioral testing procedures in the rest of the animals. General surgical procedures were the same as for MEC/CA3 lesions. One week after FG injection, the rats were perfused intracardially with saline followed by fresh 4% paraformaldehyde (PFA) in phosphate buffer (*pH* = 7.4). The brain was extracted, post-fixed overnight in 4% PFA and cryoprotected in 30% sucrose containing phosphate buffer. The brains were sectioned horizontally (40 μm) on a cryostat and thaw-mounted onto gelatin-coated slides. The FG injection site was identified as a large deposit of FG, whereas retrograde labeling is indicated by lysosomal labeling such that the cell bodies appear speckled with gold.

### Tissue preparation and processing for histology

**Figure 2:**
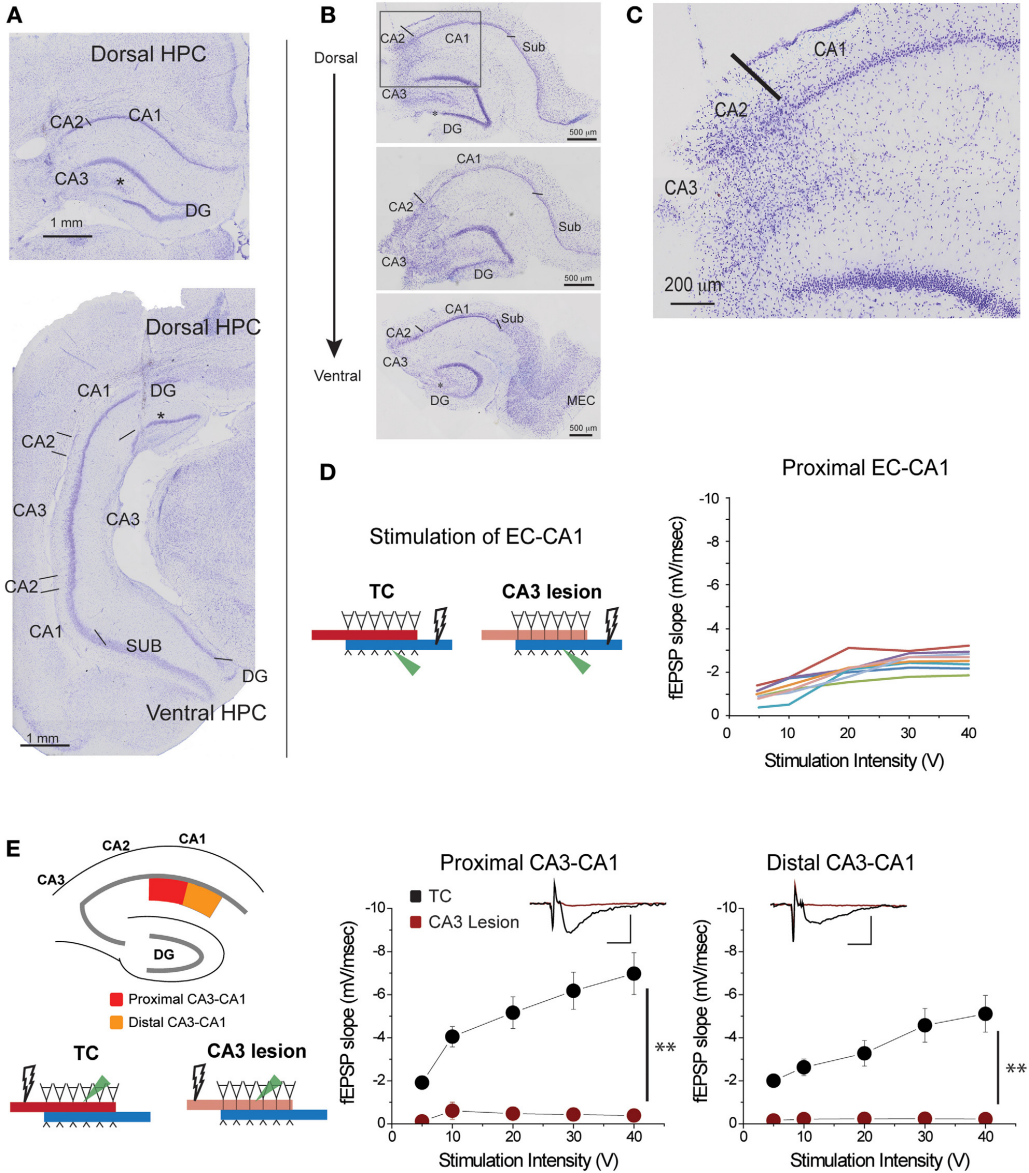
**CA3 lesions abolished CA3-CA1 transmission. (A)** Coronal sections of rat brain displaying lesions in which CA3 cells were removed from both the dorsal (top) and ventral (bottom) portions of the hippocampus. CA2 was largely spared; however, a portion of the dentate gyrus (DG) outer blade was also lesioned (asterisk). **(B)** Representative sections from hippocampal slices obtained from a lesioned rat at the end of electrophysiological recordings. CA3 is largely lesioned along the dorsal-ventral axis, although the lower blade of DG has some damage (asterisk) as well. Lines indicate the border between CA2 and CA3, and the border between CA1 and subiculum. **(C)** Magnified view of the area enclosed within the gray rectangle in the top panel of **(B)** showing the transition between lesioned and preserved hippocampal tissue. The CA1 field was spared. **(D)** Responses elicited from CA1 pyramidal neurons of CA3 lesioned animals upon EC-CA1 pathway stimulation were within normal range. This evidence corroborated the histological evidence that the CA3 damage did not extend to CA1. Each color represents a different slice. These data are the basis of the group results shown in **Figure 6A**. **(E)** CA3 lesions affected the Schaffer collaterals—CA1 transmission. To assess the functional effects of the damage, recording was performed in the proximal (red) and distal (orange) areas of the CA1 upon stimulation in stratum radiatum. After CA3 lesions, stimulating the proximal or distal CA3-CA1 synaptic path produced no synaptic responses. Inset: fEPSP traces (5 mV, 5 ms). Data points represent mean ± s.e.m. CA1, cornu ammonis area 1; CA2, cornu ammonis area 2; CA3, cornu ammonis area 3; SUB, subiculum; HPC, hippocampus; DG, dentate gyrus.

One rat with a CA3 lesion and one rat with a MEC lesion were overdosed with sodium isoflurane and perfused transcardially with normal saline, then 10% formalin (see Supplementary Table 1). Coronal sections (for the rat with the CA3 lesion) and horizontal sections (for the rat with the MEC lesion) (40 μm) were cut on a cryostat and stained with cresyl violet to evaluate the extent of the lesion. The brain tissue from the rest of the rats (4 CA3 lesion, 5 MEC lesion) was not perfused (since it was prepared for slice physiology), but post-fixed directly in 10% formalin, sectioned (for MEC, tissues was sectioned either sagitally or horizontally) and stained with Nissl as described above. The procedure to obtain hippocampal slices requires dissecting out the hippocampus and damages the rest of the brain, rendering the tissue unsuitable for regular histological examination. We solved this problem in the MEC lesioned animals by splitting the front/middle part of the brain from the back (more posterior) part of the brain. The anterior part, containing most of the hippocampus, was used for hippocampal slice physiology and the posterior part, containing the EC and some of the ventral hippocampus, was taken for EC histology studies. A trade off, however, was that the amount of tissue for EC histology was small and hence the histological data less abundant. We sectioned and stained this tissue using regular procedures. While we could assess whether the lesion encompassed the area we intended (i.e., medial EC) and spared others (e.g., lateral EC, pre- and para-subiculum), quantification *per se* was difficult because of the limited number of sections. For the CA3 lesioned group, all dorsal hippocampal slices were kept for potential electrophysiological testing to ensure efficient data collection for physiology. This meant no slice was taken fresh out of the dissection for histology. At the end of the electrophysiological experiments, during which the damage to the tissue was visible and manifested in the synaptic responses (Figures 2D,E), the untested hippocampal slices of CA3 lesioned animals (normally 1 or 2) were fixed, embedded in agarose blocks and sectioned on a vibratome (50 micrometers). Because these micro-slices come from already sliced tissue, only one or two micro-slices are optimal for histology per slice which makes quantification difficult as well as evaluation of the distance between sections.

### Delineation of regions/assessment of lesions

The hippocampal regions were delineated with the aid of the online Rat Hippocampus Atlas (Kjonigsen et al., 2011). The CA1 hippocampal subregion was differentiated from the CA2/3 and subicular subregions by the closely packed pyramidal cells. Subiculum is recognized by the abrupt broadening of the pyramidal layer. CA2 was differentiated from CA1 also by a widening of the cell layer on the proximal (close to DG) side. When possible, such as when CA3 was not lesioned, CA2 was differentiated from CA3 by the presence of smaller, densely packed cells, similar to those found in CA1, and the dispersed cells that delineated CA2 from CA1. In sagittal sections, the MEC was delineated according to Boccara et al. (2010). In both sagittal and horizontal planes, the MEC is easily identified by the characteristic packing of layer II cells. In horizontal sections, the EC is further identified by the presence of the cell-free lamina dissecans. Because lesions did not include the entire dorsal-ventral or medial-lateral extent, these characteristic cell layers could be used to identify the dorsal and ventral EC borders in both sagittal and horizontal sections. In addition, the parasubiculum was used as a local landmark to determine the medial border of MEC/parasubiculum in horizontal sections.

### Hippocampal slice recording

Transverse hippocampal slices (400 μm) were obtained from adult 4–5 months old rats: 6 Ctr, 4 TC, 4 CA3 lesion, and 7 MEC lesion (Supplementary Table 1). Because processing tissue for histological slides requires that the hippocampus be dissected out and sectioned transversally, the brain of one animal with CA3 lesion was processed for histological assessment without slice physiology (Figure 2A). Data from two of the MEC lesion animals were eventually excluded upon histological examination of the lesion. For each animal we used about 5–6 slices from the dorsal hippocampus. Slices were cut in ice cold artificial cerebrospinal fluid [ACSF containing: (mM) 119 NaCl, 4.0 KCl, 1.5 MgSO_4_, 2.5 CaCl_2_, 26.2 NaHCO_3_, 1 NaH_2_PO_4_ and 11 Glucose saturated with 95% O_2_, 5% CO_2_] and then warmed in oxygenated ACSF to 35°C for 45 min. Slices were thereafter allowed to equilibrate for at least 60 min in oxygenated ACSF at room temperature. For experiments, slices were immersed in a submerged recording chamber subfused with oxygenated ACSF at 35–36°C. *Field recordings:* Each slice was tested at all four circuits (proximal/distal CA3-CA1 and EC-CA1). The electrodes configuration was rearranged accordingly for testing of each circuit. The placing of electrodes and order of circuit testing was done so the procedure would not affect (damage) the next circuit to be tested within the same slice. A pair of stimulating (bipolar; FHC and Co, ME, USA) and recording (borosilicate glass pipette filled with ACSF; 5–10 mW) electrodes were used to evoke and record field excitatory post-synaptic potentials (fEPSP) in either the proximal or distal regions of CA1 *stratum radiatum*, where CA3 Schaffer collaterals (SC) terminate (Amaral and Witter, 1995; Witter and Amaral, 2004), or the proximal or distal regions of CA1 *stratum lacunosum-moleculare*, where EC temporoammonic (TA) projections terminate (Amaral and Witter, 1995; Witter and Amaral, 2004). Another glass pipette recording electrode (5–10 mW), placed above the fESPS recording electrode, was used to record population spikes from the CA1 *stratum pyramidale*. We therefore investigated four distinct synaptic inputs within the CA1 area: the proximal and distal portions of the SC to CA1 input (proximal and distal CA3-CA1 synapses in stratum radiatum); and the proximal and distal portions of the TA to CA1 input (proximal and distal EC-CA1 synapses in stratum lacunosum-moleculare). Test pulse duration was 50 μs. After completion of these studies, the tissue slices were placed in 10% formalin. *Statistical analysis*: Data resulting from hippocampal slice recording were plotted into input-output relationships (i.e., stimulus voltage to afferent to fEPSP slope amplitude) and analyzed with a Two-Way repeated measures ANOVA followed by a *post-hoc* Student-Newman-Keuls analysis for independent populations between series of control and lesion groups (SigmaStat 3.5; Systat Software Inc., Germany). Data points in the figures represent mean ± s.e.m.

## Results

### Lesions were localized to targeted areas

Based on histological and/or physiological assessment, the five rats in the CA3 lesion group had extensive bilateral damage to the dorsal and parts of ventral CA3 field (Figures 2A,B; Supplementary Figure 2). The damage did not extend to CA1 or subiculum fields (Figure 2C), a conclusion supported by the finding that synaptic transmission in EC-CA1 pathway of rats with CA3 lesions was at the same level as in the control group (Figure 2D). Parts of CA2 and parts of the infrapyramidal DG blade were also included in the lesion in some cases (Figures 2A–C). If the lesion effectively eliminated CA3 inputs to CA1, then there should be no CA1 synaptic response to stimulation of the CA3-CA1 pathway throughout the CA1 area in rats with CA3 lesions. We assessed stimulus-response relationships by delivering increasing electrical stimulation to the CA1 *stratum radiatum* region and recording synaptic responses within the same layer at the proximal and distal regions of area CA1 from dorsal hippocampal slices (Figure 2E). Indeed, CA3-CA1 synaptic responses were completely abolished throughout the CA1 stratum radiatum in the CA3 lesioned rats proximal CA3-CA1: TC, *n* = 15 slices; CA3 lesion, *n* = 7 slices, TC vs. CA3 lesion stimulus-fEPSP slope response curves: *F*_(1, 21)_ = 35.62, *p* < 0.001; distal CA3-CA1: TC, *n* = 15 slices; CA3 lesion, *n* = 8 slices; TC vs. CA3 lesion stimulus-fEPSP slope response curves: *F*_(1, 22)_ = 33.19, *p* < 0.001. This complete lack of transmission confirmed the effectiveness of the CA3 lesion.

**Figure 3:**
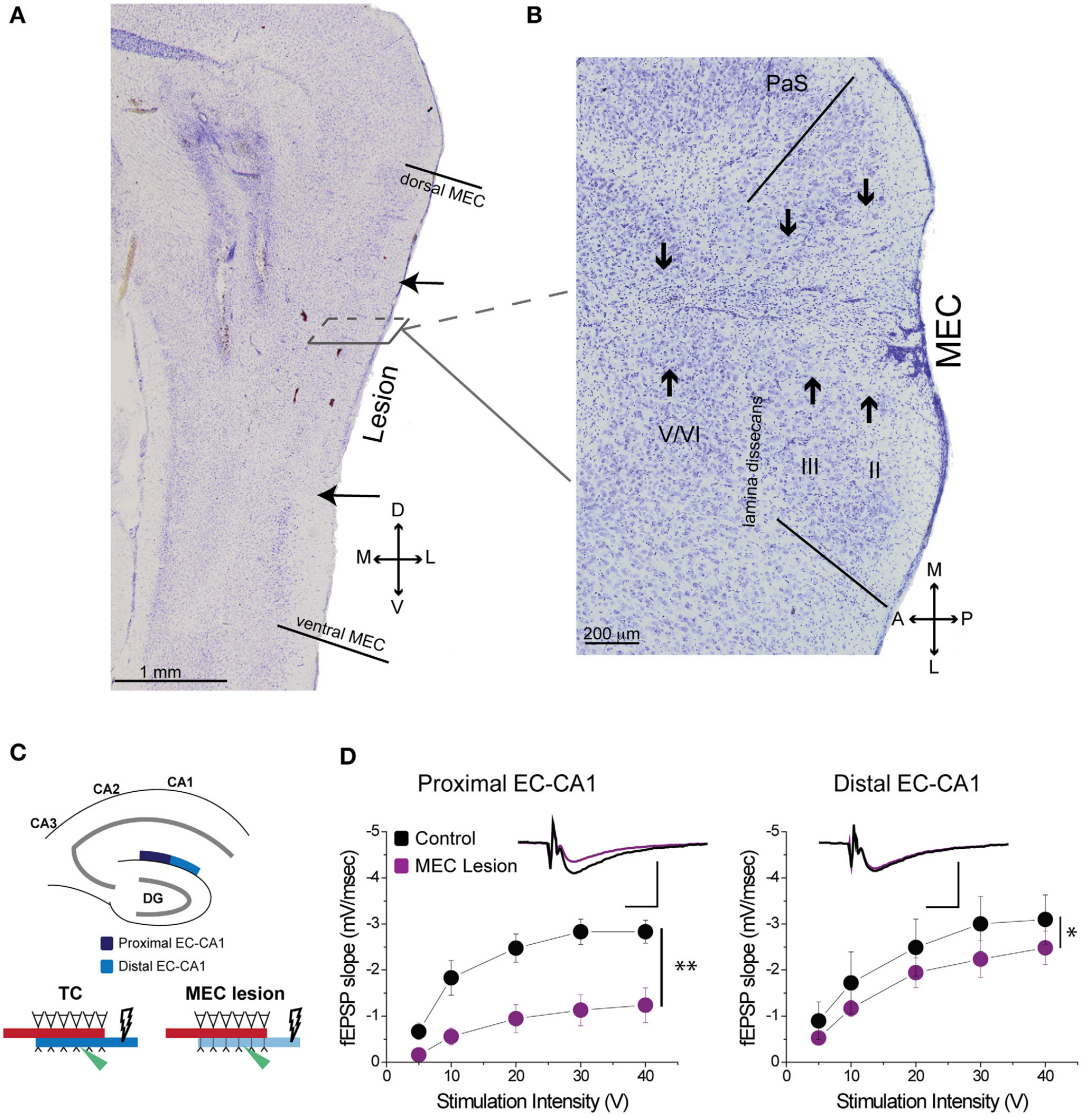
**MEC lesions diminished EC-CA1 synaptic transmission in the proximal CA1. (A)** The solid lines in the sagittal section denote the dorsal and ventral edges of MEC, while the arrows indicate the dorsal and ventral bounds of the lesion. **(B)** In a horizontal section from a different animal the solid lines demarcate the medial and lateral bounds of MEC. The arrows indicate the main lesion for each cell layer, as determined by tissue damage and cell loss. The presence of glial cells indicates that the spread of the lesion covers most of the medial-lateral extent of MEC in this case. **(C)** MEC lesions markedly affected synaptic transmission in the proximal EC-CA1 pathway. Recording was performed in the proximal (dark blue) and distal (light blue) areas of the CA1 upon stimulation in the stratum lacunosum-moleculare. **(D)** MEC lesion rats showed markedly decreased synaptic responses at the proximal EC-CA1 synaptic path (^**^) and a small decrease of synaptic responses at the distal EC-CA1 synaptic path (^*^). Inset: fEPSP traces (5 mV, 5 ms). PaS, Para subiculum; MEC, medial entorhinal cortex; and II, III, and V/VI, layers II, III, and V/VI of MEC. Data points represent mean ± s.e.m.

**Figure 4:**
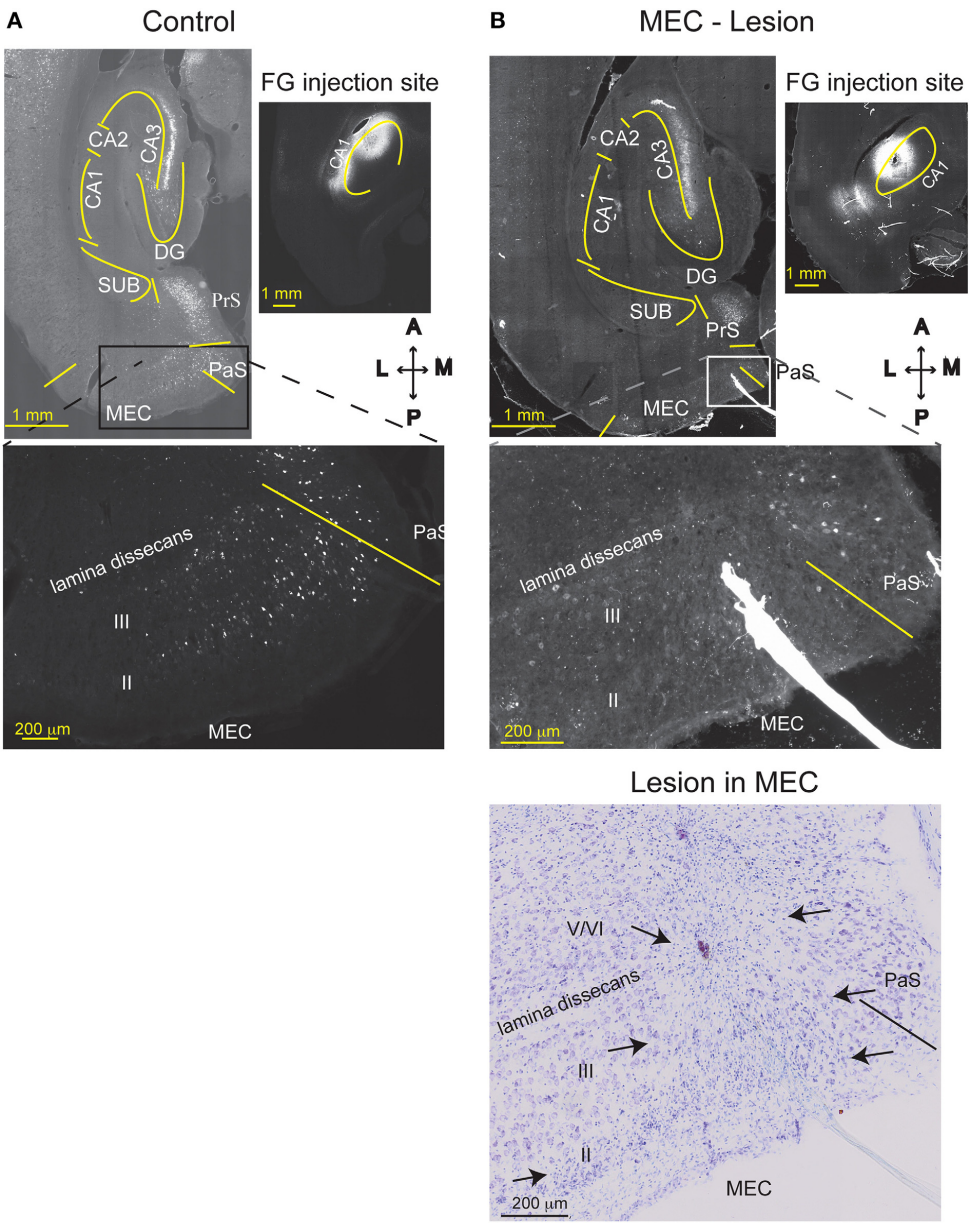
**MEC lesions were validated by loss of retrograde FG labeling**. FG was injected into comparable levels of the dorsal CA1 of a TC animal **(A)**, or a MEC lesioned animal **(B)** one week after the lesion operation occurred. In the TC animal, MEC layer III and CA3 pyramidal cells were retrogradely labeled with FG, as expected. Strong labeling is also visible in the presubiculum (PrS). Creating an excitotoxic lesion in MEC, however, resulted in attenuated labeling of MEC layer III cells. As expected, cells in CA3 were still labeled, indicating that lack of MEC label is not due to poor FG transport. The Nissl stain of the section is shown in the lower right. MEC starts at the solid line and arrows indicate the medio-lateral extent of the lesion (layers II, III, and top of layer V). CA1, cornu ammonis area 1; CA3, cornu ammonis area 3; DG, dentate gyrus; SUB, subiculum; HPC, hippocampus; PaS, Para subiculum; MEC, medial entorhinal cortex; and II, III, and V/VI, layers II, III, and V/VI of MEC. Delineations of the areas were based on the Nissl stains of the sections.

Based on histological assessment of the MEC lesions (Figures 3A,B; Supplementary Figure 3), out of the initial eight animals, we excluded two subjects from behavioral and physiological analyses. In the first rat, we found that lesion in the right hemisphere did not target MEC adequately. In the second rat, the lesion in the right hemisphere was located in the deep layers of MEC (which do not contribute a large input to the hippocampus) and included a large portion of the subiculum, while in the left hemisphere the lesion encompassed a large portion of parasubiculum. The rest of the subjects (*n* = 6) had lesions restricted to MEC that included mostly superficial layers II/III and occasionally the deep layers. In addition, the parasubiculum was sometimes damaged by the cannula passing through the tissue. To assess the impact of the MEC lesions we measured EC-CA1 synaptic transmission in hippocampal slices by evaluating the slope of fEPSPs elicited in the *stratum lacunosum-moleculare* (Figures 3C,D). We found strongly decreased synaptic responses at the proximal EC-CA1 inputs, a finding consistent with the dysfunction of abundant MEC inputs to this region [TC, *n* = 9 slices; MEC lesion, *n* = 10 slices; TC vs. MEC lesion stimulus-fEPSP slope response curves: *F*_(1, 18)_ = 35.51, *p* < 0.001]. In contrast, we found no significant changes at the distal EC-CA1 synaptic path [TC, *n* = 9 slices; MEC lesion, *n* = 10 slices; TC vs. MEC lesion stimulus-fEPSP slope response curves: *F*_(1, 18)_ = 7.23, *p* = 0.07]. Collectively, these data confirmed the effectiveness and specificity of the MEC lesions. Because stimulation in the stratum lacunosum-moleculare elicited synaptic responses in the MEC-CA1 pathway, we additionally verified the effect of the MEC lesions with anatomical tracing. To do this, we injected FluoroGold (FG) in the CA1 field of separate control and MEC lesioned rats. Properly placed MEC lesions should eliminate the FG retrograde transport from CA1 to the MEC due to loss of afferent MEC-CA1 fibers. Indeed, in control rats, labeled cells were found in the superficial layers of EC and in CA3, as expected, indicating successful injection of FG into CA1 and successful transport of FG in the brain (Figure 4A). In contrast, in a MEC lesioned rat, injection of FG into CA1 resulted in minimal labeling in MEC, but labeling in CA3 (Figure 4B), an internal positive control indicating that the lack of labeling in MEC was due to the lesion and not to lack of tracer transport. Presubiculum was labeled in both the control and MEC lesioned animals, likely due to the injection including other regions such as dorsal presubiculum (sometimes referred to as post-subiculum).

### CA3 lesions impaired spatial navigation more than MEC lesions

**Figure 5:**
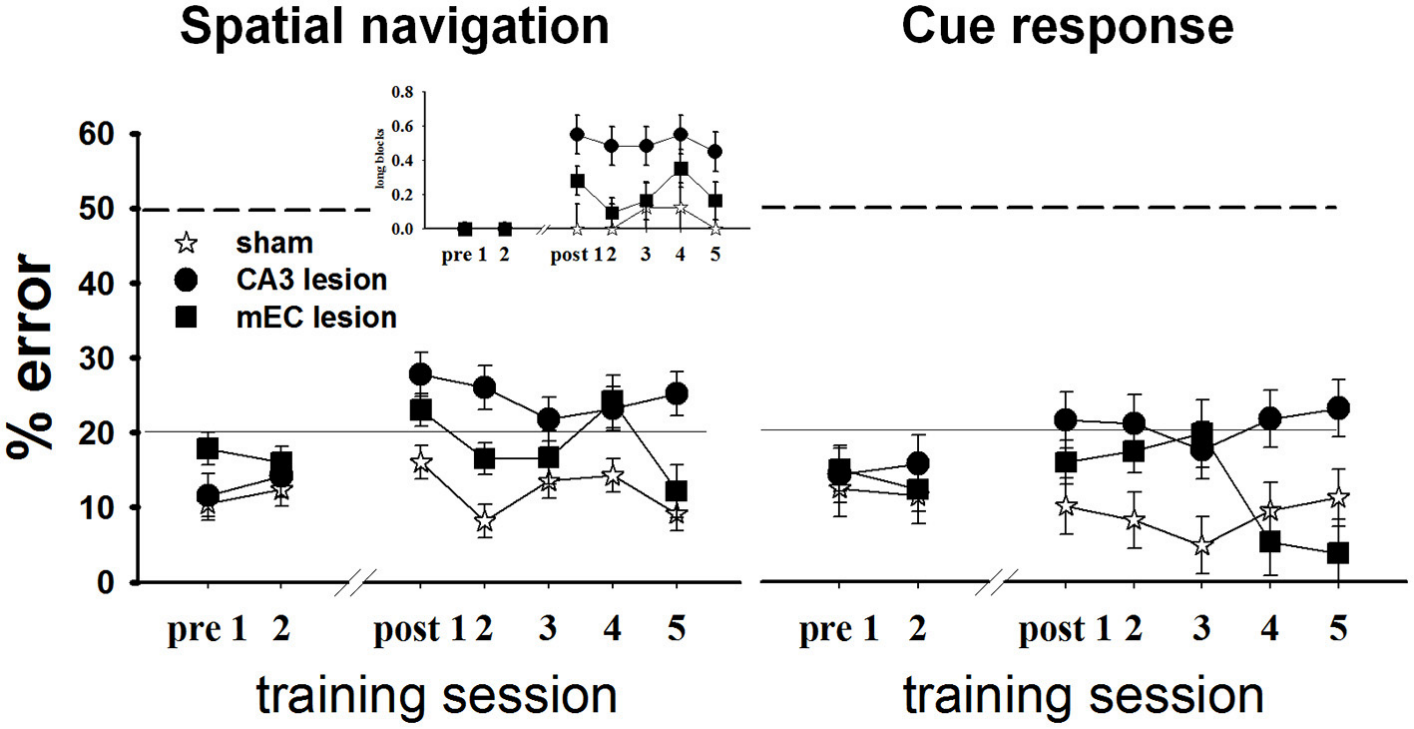
**CA3 and MEC are involved in supporting spatial navigation and cue approach performance**. Retention testing comprised all rats for 2 days (*n* = 4 TC, 5 CA3, and 6 MEC rats), and extended to 5 days for a subgroup (*n* = 4 TC, 5 CA3, and 2 MEC rats). In the spatial task (left), the performance of CA3 and MEC lesioned animals was impaired by comparison to their own pre-lesion levels, and to post-lesion level of TC group. Training reduced the behavioral deficit of the MEC lesion group but not of the CA3 lesion group. The more severe and permanent effect of CA3 lesions is well highlighted by the higher number of trials these rats needed to be switched to the next trial block throughout testing (inset). Notably, the lesions also affected the cue response (right). However, in this case the performance of the lesioned rats did not deteriorate when compared to pre-lesion levels. Dashed lines at the 50% mark indicate random level performance. Solid lines at the 20% mark indicate the criterion level each animal had to reach before surgery. Data points represent mean ± s.e.m.

To investigate the roles of CA3 and MEC in spatial information processing, we tested the effects of neurotoxic lesions of these areas on performance in a +maze alternation task that requires the animal to permanently monitor his own position relative to a spatially variable goal (Figure 5, left). During each testing day, we recorded for each rat the proportion of errors, defined as turns in the empty goal arm. All rats were trained to a criterion of 80% or better performance for 2 consecutive days before being divided into three groups and undergoing surgical procedures: CA3 lesions, MEC lesions, and sham surgery (TC group). Approximately 1 week post-lesion, we assessed performance in all rats for 5 consecutive days, except for four MEC animals who reached pre-surgery criterion after 2 days; thus only the data of the MEC animals with the worst performance were included for the last 4 days of testing. The results showed that both lesions impaired spatial performance, but compared to CA3, the effects of the MEC dysfunction were milder and temporary. The TC group maintained its performance level post-surgery [last day before vs. first day after surgery: *t*_(17.8)_ = −1.38, *p* = 0.184], and improved with extensive training [time effect across the 5 days post-surgery: *F*_(4, 12.2)_ = 5.04, *p* = 0.013]. In contrast, the performance of both lesion groups deteriorated significantly after the lesion [last day before vs. first day after surgery: CA3: *t*_(24)_ = −3.58, *p* = 0.001; MEC: *t*_(19.9)_ = −2.63, *p* = 0.016]. Training improved performance of the MEC lesion group [day1 vs. day2 post-lesion: *t*_(19.9)_ = 2.44, *p* = 0.024; time effect across the 5 days post-surgery: *F*_(4, 8.42)_ = 7.38, *p* = 0.007] but not of the CA3 lesion group [day1 vs. day2 post-lesion: *t*_(24)_ = 0.47, *p* = 0.6399; time effect across the 5 days post-surgery: *F*_(4, 15.8)_ = 1.09, *p* = 0.396]. At the end of post-lesion training (day 5 post in Figure 5), CA3 group performed worse than TC [*t*_(46.9)_ = 4.37, *p* < 0.0001] and MEC [*t*_(47.5)_ = 2.83, *p* < 0.007], while the MEC lesion group had recovered normal performance [MEC vs. TC day 5: *t*_(36.6)_ = 0.72, *p* = 0.47]. *Post-hoc* power analyses revealed that the power to detect the observed overall main effect between CA3 and TC groups, the observed main effect between MEC and TC, and the observed differences between MEC and CA3 groups during day 5, post-lesion were 99.4, 83.3, and 80.9%, respectively. The effect sizes corresponding to the above power calculations, respectively, 3.6, 2.45, and 1.72, are conventionally considered very large (Cohen, 1988).

When the location of the food was switched at the end of one trial block to the other goal arm, both lesion groups changed their choice of goal arms, but also made inappropriate turns after several correct runs to the new location (i.e., they went back to the old location). We quantified this behavior by computing the proportion of *long trial blocks*, defined as blocks of trials on which the animal did not reach criterion in 15 total runs or less, for each day (Figure 5 left, inset). CA3 lesioned animals needed more trials than TC to reach switch criterion both immediately after the surgery [*t*_(16.3)_ = 2.98, *p* = 0.008] and after extensive training [*t*_(16.3)_ = 2.44, *p* = 0.026], whereas the MEC lesion group performed at a level intermediate between CA3 and TC groups (MEC group showed no significant differences from TC or CA3 groups during either day 1 or day 5 post-lesion). Collectively, these data confirmed that CA3 and MEC neural activities are involved in spatial memory. They also suggest that the effect of the MEC lesions on spatial navigation is milder than the effect of CA3 lesions, and can be reversed through training. In contrast, CA3 lesions seem to introduce more severe and permanent effects.

### CA3 and MEC lesions affected performance in the cue-response task

We wanted to compare the performance of the lesioned animals on a task that would be memory-based and entail similar overt motor behavior but that would not involve hippocampal activity. We therefore assessed performance of all subjects in Set2 (see *Methods* and Supplementary Table 1) in a habit-type response to a visible cue, thought to be dependent on the dorsal striatum (Packard et al., 1989; McDonald and White, 1993; Packard and McGaugh, 1996; Ferbinteanu et al., 2011). Specifically, the rats had to walk toward an intramaze visual cue whose spatial location was rendered irrelevant (Figure 1A, right; Supplementary Figure 1). Based on previous data (Packard et al., 1989; Packard and McGaugh, 1996; McDonald and White, 1993; Ferbinteanu et al., 2011), we expected that all three groups would perform equally well; however, this was not the case (Figure 5, right). Although all rats maintained their level of response across the recovery interval [last day before vs. first day after surgery: TC: *t*_(18.1)_ = 0.38, *p* = 0.708; CA3: *t*_(23.1)_ = −1.30, *p* = 0.207; MEC: *t*_(19)_ = −1.17, *p* = 0.257], the CA3 lesion group performed worse than TC immediately after the lesion [day 1 post-lesion: *t*_(37.1)_ = 2.23, *p* = 0.031] and after extensive training [day 5 post-lesion: *t*_(38.9)_ = 2.33, *p* = 0.025]. The MEC lesioned rats were worse than TC only during day 3 [*t*_(37.1)_ = 2.63, *p* = 0.012], but significantly better than the CA3 group at the end of testing [day5 post-lesion: *t*_(45.9)_ = 3.29, *p* = 0.002]. Since we consider the results for cue responses as exploratory, we did not conduct an in-depth *post-hoc* power analysis. Nevertheless, the effect size for the main effect between CA3 and TC groups was 1.32; the effect size for the main effect between MEC and controls was 0.58, and the effect size between lesions at day 5 was 1.99. These effect sizes, although not as large as the corresponding effect sizes for spatial navigation, are still significant (Cohen, 1988). Thus, the data suggest that lesions of the hippocampal system may result in impairment of behavior otherwise thought to be hippocampal-independent.

### MEC lesions and extensive training enhanced CA3 input to proximal CA1

**Figure 6:**
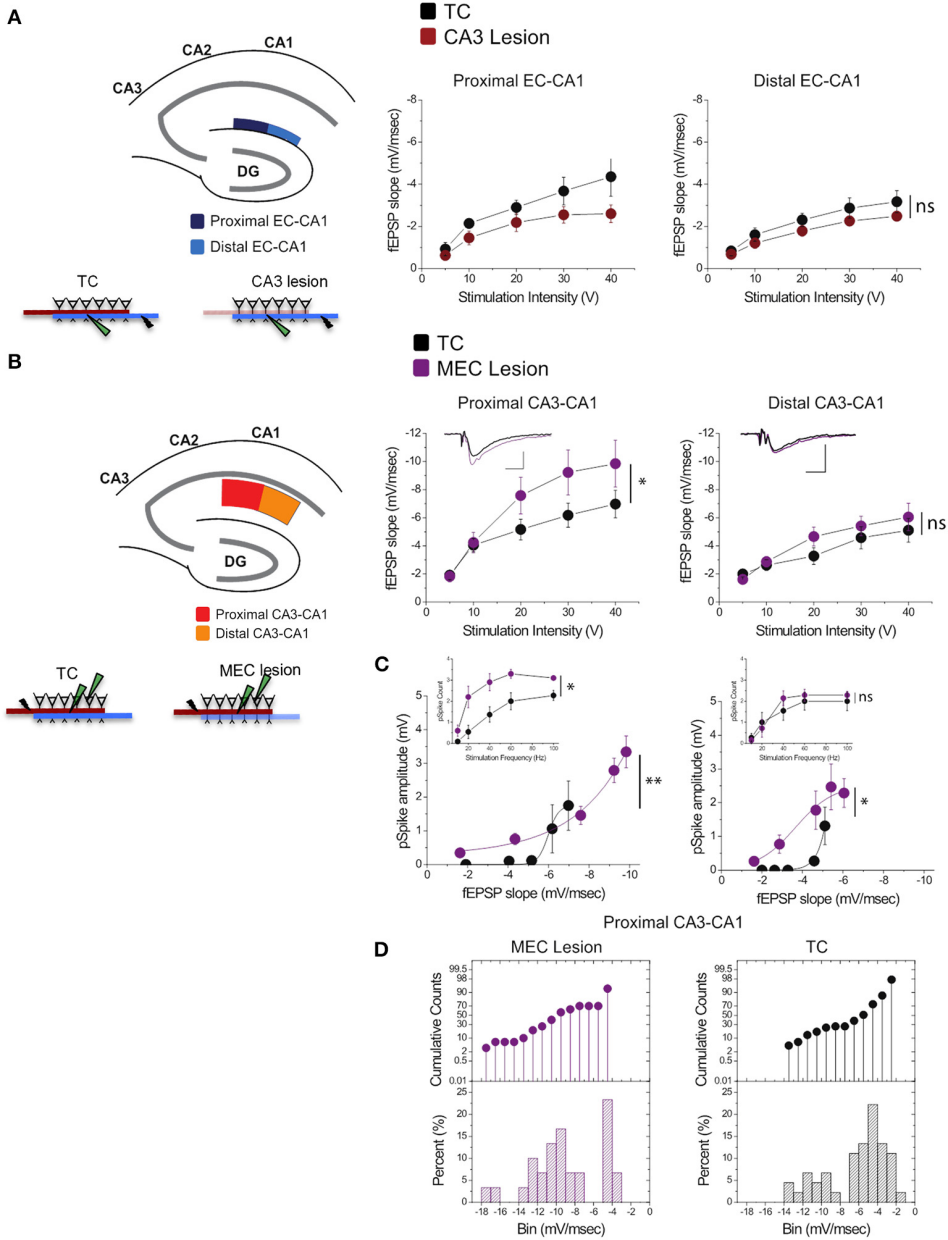
**MEC lesions selectively enhanced CA3-CA1 synaptic transmission. (A)** CA3 lesions did not affect transmission in the MEC-CA1 pathway. In CA3 lesioned rats, stimulation and recording were performed in the proximal (dark blue) and distal (light blue) areas of the CA1 stratum lacunosum-moleculare, site of the EC-CA1 inputs The results showed no differences in synaptic transmission across all levels of stimulation between lesioned and TC groups. Data points represent mean ± s.e.m. **(B)** MEC lesions markedly increased CA1 synaptic responses (^*^). Stimulation and fEPSP recording were performed in the proximal (red) and distal (orange) areas of the CA1 stratum radiatum (top panels). Compared to TC, MEC lesion rats showed a two-fold increase of synaptic responses at the proximal CA3-CA1 synaptic path and restricted increase in synaptic responses at the distal CA3-CA1 synaptic path. Inset: fEPSP traces (5 mV, 5 msec). Data points represent mean ± s.e.m. **(C)** The activity of CA1 neurons driven by synaptic responses evoked at the proximal CA3-CA1 path was remarkably increased in MEC lesioned rats (^**^). Population spike (pSpike) recording was performed in the CA1 stratum pyramidale upon stimulation in the proximal (red) and distal (orange) areas of the CA1 stratum radiatum. Inset: The number of pSpikes evoked by increasing frequency of stimulation at the proximal CA3-CA1 path was also increased in MEC lesion rats (^*^). A similar but smaller effect was present at the distal CA3-CA1 in these rats (^*^). Note the higher fEPSP slope of the MEC lesioned group. Data points represent mean ± s.e.m. **(D)** MEC lesions increased the fEPSP slope throughout the dorsal hippocampus. The cumulative counts (top) and the percentage (bottom) of slice recordings within given ranges (bins) of fEPSP slope amplitude show that almost 70% of the recorded slices from MEC lesion rats distribute mostly over higher fEPSP slope amplitudes (between −8 and −14 mV/ms; left) compared to the TC group that distribute mostly between −2 and −6 mV/ms than between −8 and −14 mV/ms (right).

Stimulation of perforant path inputs to hippocampal CA1 pyramidal neurons induces long-term potentiation at the Schaffer collaterals-CA1 synapses when the first precedes the second by 20 ms (Dudman et al., 2007). Therefore, we were interested in how lesion in one of the pathways may modify synaptic transmission in the other. We therefore assessed synaptic strength in EC-CA1 pathway in TC and CA3 lesioned groups, and CA3-CA1 synaptic strength in TC and MEC lesioned groups. CA3 lesions that abolished CA3-CA1 synaptic function did not alter substantially EC-CA1 synaptic transmission [Figure 6A; proximal EC-CA1: TC, *n* = 9 slices; CA3 lesion, *n* = 7 slices, TC vs. CA3 lesion effect only at 40 V stimulus: *F*_(1, 15)_ = 15.04, *p* < 0.05; distal EC-CA1: TC, *n* = 9 slices; CA3 lesion, *n* = 9 slices, TC vs. CA3 lesion stimulus-fEPSP slope response curves: *F*_(1, 17)_ = 3.07, *p* = 0.07]. In contrast, MEC lesions that impaired the MEC-CA1 synaptic responses markedly enhanced synaptic transmission in the proximal CA3-CA1 path [Figure 6B; proximal CA3-CA1: TC, *n* = 15 slices; MEC lesion, *n* = 8 slices; TC vs. MEC lesion stimulus-fEPSP slope response curves: *F*_(1, 22)_ = 16.40, *p* < 0.01; distal CA3-CA1: TC, *n* = 15 slices; MEC lesion, *n* = 7 slices, TC vs. MEC lesion stimulus-fEPSP slope response curves: *F*_(1, 21)_ = 2.83, *p* = 0.076]. The enhanced CA3-CA1 synaptic transmission observed in MEC lesion rats translated into increased amplitude [TC, *n* = 15 slices; MEC lesion, *n* = 9 slices, TC vs. MEC lesion fEPSP-pSpike amplitude curves: *F*_(1, 23)_ = 23.81, *p* < 0.001] and number of population spikes [TC, *n* = 15 slices; MEC lesion, *n* = 9 slices; TC vs. MEC lesion stimulus frequency-pSpike number count: *F*_(1, 22)_ = 18.23, *p* < 0.01] recorded in the pyramidal layer of CA1 area upon CA3-CA1 input stimulation (Figure 6C), suggesting increased excitability of CA1 principal cells. A similar but smaller effect was present for the amplitude of pSpike in CA1 neurons by stimulation of the distal CA3-CA1 inputs [TC, *n* = 15 slices; MEC lesion, *n* = 7 slices, TC vs. MEC lesion stimulus fEPSP-pSpike amplitude curves: *F*_(1, 21)_ = 13.46, *p* < 0.05], but not for the number of pSpikes [TC, *n* = 15 slices; MEC lesion, *n* = 7 slices; TC vs. MEC lesion stimulus frequency-pSpike count curves: *F*_(1, 21)_ = 5.36, *p* = 0.21]. The impact on the proximal CA3-CA1 synaptic path was consistent across slices from the dorsal hippocampus. About 70% of the recorded slices from MEC rats showed heightened synaptic responses compared to TC rats (Figure 6D), suggesting an indirect but strong relationship between the MEC-CA1 projection and CA3-CA1 synaptic function.

**Figure 7:**
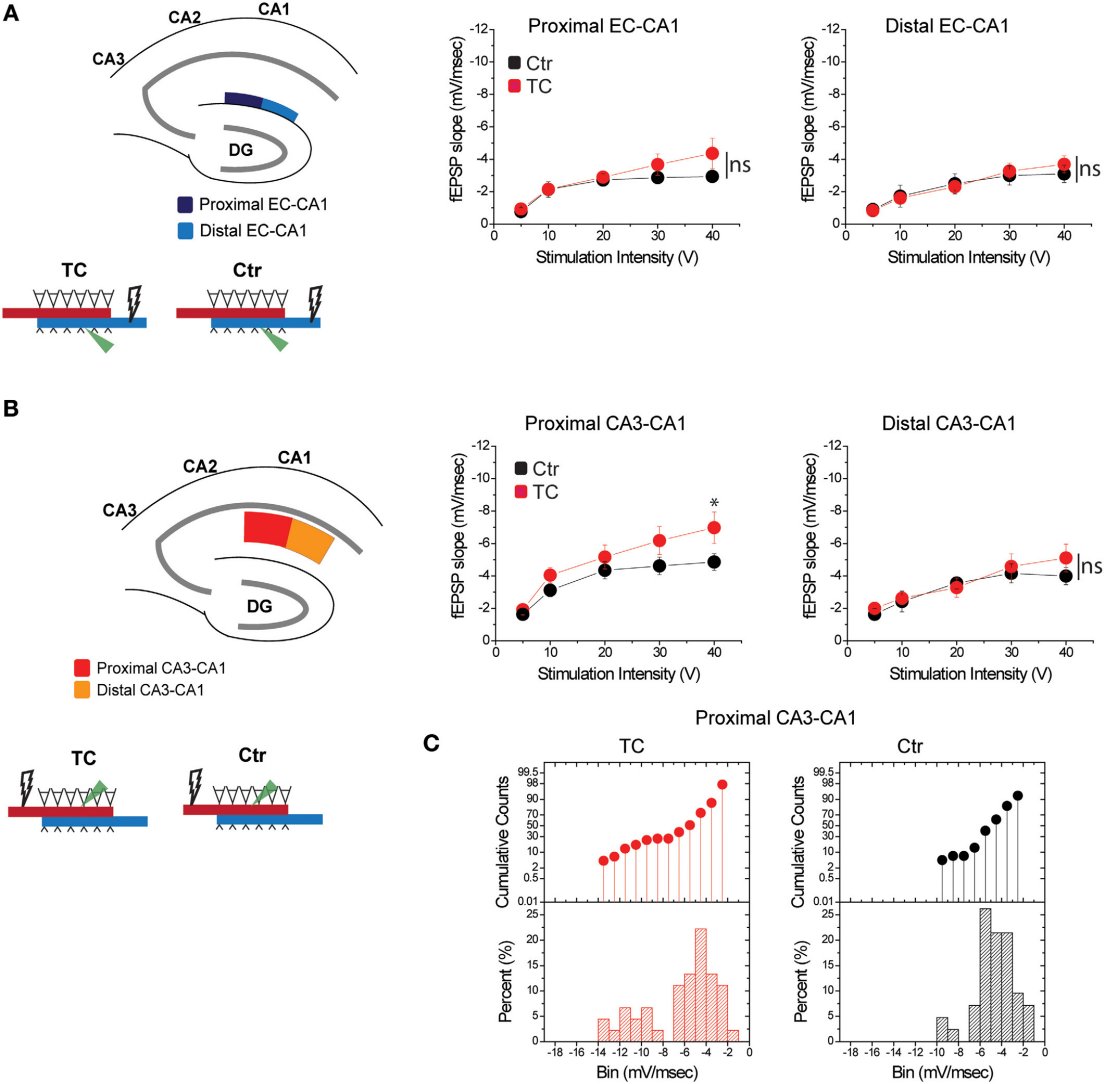
**Modulation of CA3-CA1 synaptic transmission in TC rats. (A)** Stimulation and recording were performed in the proximal and distal areas of stratum lacunosum-moleculare (site of EC-CA1 connections). Training did not affect synaptic transmission in the EC-CA1 path. Data points represent mean ± s.e.m. **(B)** Stimulation and recording were performed in the proximal and distal areas of stratum radiatum (site of CA3-CA1 connections) in Ctr and TC rats. Training significantly enhanced synaptic response in the proximal (^*^) but not distal CA3-CA1 path. Data points represent mean ± s.e.m. **(C)**. The cumulative counts (top) and the percentage (bottom) of slice recordings within given ranges (bins) of fEPSP slope amplitude in the proximal CA3-CA1 synaptic path show that about 20% of recorded slices from TC rats distribute over higher fEPSP slope amplitudes (left) compared to the Ctr group that mostly distribute between −2 and −6 mV/ms (right). The distribution of fEPSP amplitude across slices is representative of each individual animal.

To distinguish the effects of lesions from the effects of extensive training, we compared synaptic function in all paths (CA3-CA1 and EC-CA1, proximal and distal) in dorsal hippocampus slices from TC rats vs. control rats (Ctr) that did not undergo the extensive training. We detected no changes in synaptic response in the EC-CA1 pathway [Figure 7A; synaptic responses in the proximal EC-CA1 path: Ctr, *n* = 13 slices; TC *n* = 9 slices; Ctr vs. TC stimulus-fEPSP slope response curves: *F*_(1, 20)_ = 2.4, *p* = 0.14; synaptic responses in the distal EC-CA1 path: Ctr, *n* = 9 slices; TC, *n* = 9 slices; Ctr vs. TC stimulus-fEPSP slope response curves: *F*_(1, 16)_ = 2.7, *p* = 0.12], but we found a modest enhancement of CA3-CA1 synaptic transmission in the proximal CA1 region of TC rats (Figure 7B). The effect was statistically significant only at 40 V of stimulation in the proximal CA1 [Ctr, *n* = 13 slices; TC, *n* = 15 slices; Ctr vs. TC at 40 V, difference between means = 0.02, *p* = 0.02; Ctr vs. TC stimulus-fEPSP slope response curves: *F*_(1, 20)_ = 2.25, *p* = 0.15]. No significant effect on synaptic responses was found at the distal CA3-CA1 synaptic path [Ctr, *n* = 9 slices; TC, *n* = 14 slices; Ctr vs. TC stimulus-fEPSP slope response curves: *F*_(1, 21)_ = 2.36, *p* = 0.1]. The CA3-CA1 enhancement effect was present in 20% of the slices recorded from TC rats compared to control rats (Figure 7C). The small effect on CA3-CA1 synaptic function observed in TC rats confirmed our result that MEC area dysfunction has a large impact on CA3-CA1 synaptic function, and also highlights that extensive training allowed a modest but detectable readout of the comparatively localized role of learning on the modulation of synaptic function in the dorsal hippocampus.

## Discussion

Our paper is framed within the memory systems theory, which emphasizes that distinct memory representations are formed through activity in distinct brain circuits. The hippocampal system has a highly topographic anatomical organization which influences physiology, and this in turn is relevant for behavior. We investigated the relative contributions of CA3 and MEC to performance of spatial navigation and cue-response tasks by testing rats with neurotoxic NMDA lesions of these structures. Evaluation of the neural damage combined histological and physiological methods. As predicted by the topographical organization of anatomical connections in the hippocampal formation, CA3 lesions abolished information flow in the CA3-CA1 pathway and MEC lesions weakened transmission of the EC input to proximal CA1. Both CA3 and MEC lesions impaired spatial navigation, but the latter had milder and temporary effects compared to the former. Notably, the lesions also affected nonspatial navigation based on the ability to follow a visible cue, a behavior considered to reflect striatal rather than hippocampal function. MEC lesions greatly increased transmission in the proximal CA3-CA1 pathway, while extended behavioral training had a similar, although much more restricted effect. Thus, heterosynaptic interaction within the CA1 local circuits may recruit some of the neural processes typically involved in learning to compensate for damage to cortical input.

Our data show that comparative to CA3 lesions, the effects of MEC lesions on spatial navigation are milder and temporary. While numerous other behavioral and electrophysiological studies document the involvement of MEC and CA3 in the processing of spatial information (Miller and Best, 1980; Sutherland et al., 1983; Jarrard et al., 1984; Schenk and Morris, 1985; Quirk et al., 1992; Good and Honey, 1997; Frank et al., 2000; Oswald and Good, 2000; Fyhn et al., 2004; Lee and Kesner, 2004; Leutgeb et al., 2004; Parron et al., 2004; Remondes and Schuman, 2004; Hargreaves et al., 2005; Steffenach et al., 2005; Alvernhe et al., 2008; Tort et al., 2009; Gaskin and White, 2010; Mizuseki et al., 2012), our study is the first to directly compare the functional importance of MEC input and intrahippocampal CA3 processing for spatial behavior. This result, consistent with the findings that lesions of the EC do not abolish the formation of place fields in the CA1 neurons (Brun et al., 2008; Van Cauter et al., 2008) and that CA3 lesions reduce the spatial selectivity of CA1 activity (Brun et al., 2002), emphasizes that unlike cortical input to CA1, damaged intrahippocampal processing cannot be functionally compensated (cf. Sutherland et al., 1983, 2001).

Several factors can lead to the mild character of the behavioral impairments. First, unlike complete/massive obliteration of the hippocampus (via lesion or genetic manipulations), partial/small manipulations most likely show none to mild significant behavioral changes. A remarkable case is Tonegawa's group demonstration that selective ablation of NMDA receptor in area CA3 affects pattern completion processes (Nakazawa et al., 2002). The deficit was only revealed by modulating the presentation of the spatial cues in a modified spatial task. Other examples include our own results with dorsal vs. ventral hippocampal lesions (Ferbinteanu et al., 2003). The later produce a mild spatial deficit likely due to the less spatial nature of the input to, and properties of information processing in this area (Amaral and Witter, 1995; Witter and Amaral, 2004; Kjelstrup et al., 2008). Here, we only lesion parts of the hippocampus (CA3) or its input (MEC). Slice recordings demonstrate that remaining parts of the hippocampal circuitry remain functional and this activity (spatial input from LEC transmitted to the hippocampus proper in MEC lesioned animals, Knierim et al., 2014; and direct EC-CA1 input in CA3 animals) likely is involved in spatial performance in the present experiment. Second, behavioral performance can be supported by multiple memory systems and severe deficits occur only after all memory systems are damaged, as demonstrated by McDonald and White (1995) in their study of hippocampal and nonhippocampal contributions to place learning. A similar situation may be the case in the current experiment. An ongoing study in our lab suggests that both the spatial and cue response tasks involve the activity of dorsal striatum regardless of whether the tasks are acquired concurrently or in parallel.

An alternative interpretation of our current data is that the stronger effect of CA3 may be originating in damage to the infrapyramidal blade of the DG, an area also involved in spatial navigation (McNaughton et al., 1989; Hunsaker and Kesner, 2008; Morris et al., 2012). Three factors argue against this possibility. First, damage to the DG was partial: the upper blade of the DG was not affected, while lesions to the lower blade of the DG were incomplete (Figure 2A). Second, DG seems to be involved primarily during early stages of spatial memory formation (Lee and Kesner, 2004; Jerman et al., 2006; Poirier et al., 2008; Schlesiger et al., 2013), while our lesioned animals performed tasks well learned prior to surgery. Third, our MEC lesions also interfered with DG input to some extent, as these lesions often included layer II EC neurons that project directly to the granule cells (Witter and Amaral, 2004). Thus, the behavioral effects of MEC may also have reflected some degree of DG dysfunction. Therefore, partial DG damage is unlikely to be the cause of differences between the effects of CA3 and MEC lesions on behavior. On the other hand, if some of the spatial impairment of MEC lesioned animals can be attributed to damage of the MEC-CA3 input, then it would further argue for a limited role of MEC-CA1 input.

Here we found that lesions of the hippocampal network also impaired navigation based on a visual cue, a cognitive function thought to be independent of hippocampal activity (McDonald and White, 1994; Packard and McGaugh, 1996). The results of the cue-response task were contrary to our prediction but provide a *post-hoc* explanation of our previous electrophysiological results that in rats trained with this paradigm, a similar number of place fields are active across tasks, and similar proportions of those place fields show prospective and retrospective activity (Ferbinteanu et al., 2011). Validated by future research, these findings would have important implication for the memory systems theory because they indicate that the contribution of neural circuits to behavior is not rigidly set, but rather depends on past experience (Sparks et al., 2011a,b; McDonald and Hong, 2013). Because large hippocampal lesions do not affect performance in rats trained in nonspatial navigation based on response to the visible cue (Ferbinteanu et al., 2011), MEC or CA3 lesions are unlikely to interfere with motivation, attention, memory for the rules of the task, basic motor behavior, or ability to flexibly modify overt behavior in pursuit of a goal. Consistent with this idea, the rats in the current experiment ran reliably on the maze, entered and switched goal arms readily (with no return to the start arm), and consumed the food quickly. Thus, the critical factor that most likely explains the discrepancy between previous and current results is the training regimen. Previously, including in our own behavioral work, each rat was trained and tested in only one of the two tasks. In contrast, in the present experiment the animals had to concurrently learn a spatial navigation *and* a cue-response task set in competitive interaction (that is, only one of the two strategies was adequate at a time and the other strategy could provide conflicting behavioral guidance). An earlier study using a similar competitive setting of spatial navigation and cue-response on a radial maze similarly found that lesions of EC affected both types of memory, and that the performance deficit in the cue-response task recovered with extensive training (Jarrard et al., 1984). In that study, the absence of recovery in the spatial task may be related to the electrolytic nature of the lesions, which eliminate both cell bodies and fibers of passage. The matching experiment investigating the role of CA3 in a similar paradigm used unilateral damage (Jarrard, 1983) and thus the data cannot be directly compared to the study we present here. Nonetheless, the results of this earlier study provide evidence that damage to the hippocampal system can interfere with cue-response when training encompasses concurrent acquisition of two different strategies. It is also conceivable that partial damage to the hippocampal system may cause behavioral deficits not seen with complete hippocampal lesions. In this alternative, a “defective” spatial strategy may still compete and impede behavioral control of the cue-response strategy. Further research will have to investigate these alternatives.

Our pattern of results prompts an important question regarding the relative contributions of hippocampal cortical input and intrahippocampal processing to spatial memory. If the MEC is the source of spatial input to the hippocampus and forms a metric representation of the animal's position in the environment (Moser and Moser, 2008; Buzsaki and Moser, 2013) then how do rats with MEC lesions find their way in various spatial tasks (e.g., plus maze: current experiment; radial maze: Pouzet et al., 1999); water maze (Burwell et al., 2004; Steffenach et al., 2005; Van Cauter et al., 2013); delayed matching-to-position (Pouzet et al., 1999)? One possibility is that activity in spared dorsocaudal MEC may be sufficient to support spatial behavioral function (cf. Steffenach et al., 2005). If so, spatial deficits should become severe and permanent when lesions encompass this area. Empirical results however indicate that rats with MEC lesions that include the dorsocaudal area are only mildly impaired and/or demonstrate ability to learn in spatial tasks (Burwell et al., 2004; Steffenach et al., 2005; Van Cauter et al., 2013). A second possibility is that spatial information continues to reach the hippocampus from the LEC (Hunsaker et al., 2013; Van Cauter et al., 2013; Knierim et al., 2014) and may be sufficient to support spatial navigation. Through similar logic, damage of the entire EC (comprising MEC and LEC) should then be associated with severe and permanent spatial deficits. Although it is likely that LEC does convey spatial input to the hippocampus, empirical results in experiments targeting the entire EC do not support this hypothesis either (Schenk and Morris, 1985; Good and Honey, 1997; Pouzet et al., 1999; Oswald and Good, 2000; Oswald et al., 2003; Jarrard et al., 2004).

A third possibility is that the preserved spatial abilities in animals with MEC damage involve the facilitated transmission in the proximal CA3-CA1 pathway. Since no previous studies have followed an approach similar to ours, the generality of this novel finding cannot currently be evaluated, but suppression of activity through administration of tetrodotoxin (TTX) in rats (Echegoyen et al., 2007) and in cultured hippocampal systems (Turrigiano et al., 1998; Kim and Tsien, 2008) resulted in hyperexcitability of CA1 neurons upon stimulation of Schaffer collaterals, and enhanced CA3-CA1 synaptic transmission, respectively. Remarkably, the lesion-induced process may recruit some of the same mechanisms involved in learning (Turrigiano, 2008). We found facilitated synaptic transmission in the CA3-CA1 pathways of our rats subjected to extensive behavioral training, a finding corroborated by results obtained with trace eyeblink conditioning in rabbits (Moyer et al., 1996) and mice (Gruart et al., 2006); operant conditioning in mice (Jurado-Parras et al., 2013); and inhibitory avoidance (Whitlock et al., 2006) and object recognition (Clarke et al., 2010) in rats. Burst activation of the EC-CA1 pathway enhances CA3-CA1 synaptic transmission (Remondes and Schuman, 2002; Han and Heinemann, 2013), which in turn leads to greatly enhanced long-term potentiation (Han and Heinemann, 2013), a plasticity mechanism involved in normal learning (Whitlock et al., 2006). Thus, in normal animals, facilitated CA3-CA1 information flow prompted by strong EC-CA1 input may support behavioral learning (Han and Heinemann, 2013). In animals with damaged EC, the large increase in CA3-CA1 synaptic transmission may support much more efficient information processing of diminished cortical input, sufficient to maintain the spatial specificity and/or prevent the post-lesion degradation of CA1 activity (Brun et al., 2002, 2008). Conceivably, this phenomenon in turn would render the associated behavioral spatial deficits mild and temporary.

The underlying mechanism of the heterosynaptic interactions between CA3-CA1 and EC-CA1 pathways may be complex and temporal factors likely play an important role. High frequency stimulation delivered in the stratum lacunosum-moleculare of area CA1, which receives the direct projections from layer III EC, prevents CA1 neurons from responding to Schaffer collaterals input (Dvorak-Carbone and Schuman, 1999), but long lasting potentiation of excitatory post-synaptic potentials (EPSPs) in the CA3-CA1 pathway occurs if EC-CA1 (temporoammonic) pathway is stimulated 20 ms in advance (Dudman et al., 2007). This form of synaptic plasticity, referred to as input-timing-dependent plasticity, takes place in the absence of neural firing in CA1 field, and is induced through a pattern of activity that mimics the latency of propagation through the tri-synaptic circuit (Yeckel and Berger, 1990) and the natural properties of neural firing in the EC (Frank et al., 2001). Other factors of potential importance include: homeostatic synaptic plasticity (Turrigiano et al., 1998; Turrigiano, 2011; Vitureira et al., 2012) which, through compensatory mechanisms, leads to enhanced responses in the CA3-CA1 pathway in the absence of input to the hippocampus (Echegoyen et al., 2007; Kim and Tsien, 2008); post-lesion sprouting (Ramirez et al., 1996); and potentiation of AMPA receptor-dependent synaptic transmission and increased contribution of synaptic GluN2B (Han and Heinemann, 2013). Further experiments will be needed to clarify how interactions between circuits in the local hippocampal network contribute to learning processes.

In conclusion, our results suggest that intrahippocampal CA3 processing is critically important for normal memory-based behavior and that it can interact heterosynaptically with cortico-hippocampal input to modulate activity in the CA1. Combined with past learning experience, this modulation may have important consequences for the contribution of each circuit to activity in the CA1 field, and ultimately to memory-dependent behavior.

## Author contributions

All authors participated in the design and execution of the experiments, data analysis, and preparation of the manuscript.

### Conflict of interest statement

The authors declare that the research was conducted in the absence of any commercial or financial relationships that could be construed as a potential conflict of interest.
