# Supplementary material for: Relative contributions of CA3 and medial entorhinal cortex to memory in rats
Source: Front Behav Neurosci. 2014 Aug 28;8:292. doi: 10.3389/fnbeh.2014.00292 (PMC4148030; doi:10.3389/fnbeh.2014.00292)
Supplement: Supplementary file 1 [file Presentation1.PDF]

A

Date: Nov-20/2012 **Block Mode:** OFF

Day#: Day 9 **Rat:** CA3-5

#	Journey	Notes:	#	Journey	Notes:
1	NW	X	31	SW	✓
2	NE	✓	32	SW	✓
3	SW	✓	33	NW	✓
4	NE	✓	34	SE	✓
5	SW	✓	35	SW	✓
6	SE	✓	36	NE	✓
7	SE	✓	37	NW	X
8	NW	✓	38	SE	✓
9	NW	X	39	NE	✓
10	NW	✓	40	NW	X
11	SE	✓	41	NE	
12	NE	✓	42	SW	
13	SE	✓	43	NW	
14	SW	✓	44	SE	
15	SW	✓	45	NW	
16	NE	✓	46	SE	
17	NE	✓	47	NE	
18	SE	✓	48	SE	
19	NW	✓	49	SW	
20	NW	X	50	SW	
21	NW	✓	51	NE	
22	SE	✓	52	SE	
23	SW	✓	53	NE	
24	SW	X	54	SW	
25	NW	✓	55	NW	
26	SE	✓	56	NW	
27	SE	✓	57	SE	
28	SE	✓	58	SW	
29	NW	✓	59	SE	
30	SE	✓	60	NE	

6/40 = 15%

B

Date: Nov / 20 / 2012 **Block Mode:** ON

Day#: Day 7 **Rat:** CA3-5

3/40 = 7%

#	Journey	Notes:	#	Journey	Notes:
1	N	W X	31	S	E ✓
2	S	✓	32	S	✓
3	S	✓	33	N	✓
4	S	✓	34	N	✓
5	N	✓	35	S	✓
6	N	✓	36	S	✓
7	S	✓	37	S	✓
8	S	✓	38	N	✓
9	N	✓	39	S	✓
10	S	✓	40	N	✓
11	S	E ✓	41	N	
12	S	✓	42	N	
13	N	X	43	S	
14	N	✓	44	N	
15	S	✓	45	N	
16	N	✓	46	N	
17	S	✓	47	S	
18	N	✓	48	S	
19	S	✓	49	N	
20	S	✓	50	N	
21	N	W ✓	51	S	
22	N	✓	52	N	
23	N	✓	53	N	
24	S	✓	54	S	
25	N	✓	55	S	
26	S	X	56	N	
27	N	✓	57	S	
28	S	✓	58	S	
29	N	✓	59	S	
30	S	✓	60	N	

C

Date: ___/___/______ **Block Mode:** ON

Day#: Dec 3/2012 Day 1 **Rat:** CA3 - 5

#	Journey	Notes:	#	Journey	Notes:
1	N	W ✓	31	S	✓
2	S	✓	32	S	✓
3	S	✓	33	S	X .
4	S	✓	34	N	X .
5	N	✓	35	S	X .
6	N	X .	36	S	✓
7	N	✓	37	S	✓
8	S	✓	38	N	X .
9	S	✓	39	N	✓
10	S	✓	40	S	X .
11	N	E ✓	41	N	E ✓
12	S	✓	42	N	✓
13	N	X	43	N	✓
14	N	X	44	S	✓
15	S	✓	45	S	✓
16	S	✓	46	S	✓
17	N	✓	47	N	✓
18	N	X	48	N	✓
19	S	✓	49	S	✓
20	S	✓	50	S	✓
21	N	X	51	S	
22	S	✓	52	N	
23	S	✓	53	N	
24	N	✓	54	S	
25	N	✓	55	S	
26	N	W X .	56	S	
27	S	X .	57	N	
28	S	✓	58	S	
29	S	✓	59	N	
30	N	X .	60	N	

15 trials (rows 11–25)

15 trials

$\frac{13}{50} = 26\%$

D

Date: Dec / 3 / 2012 **Block Mode:** OFF

Day#: Day 1 **Rat:** CA3-5

#	Journey	Notes:	#	Journey	Notes:
1	SE	✓	31	SW	X
2	SE	✓	32	NW	X
3	SE	✓	33	SE	X
4	NW	✓	34	NW	✓
5	NE	✓	35	SW	X
6	SW	✓	36	NW	✓
7	NE	✓	37	NE	✓
8	SE	✓	38	NW	✓
9	NW	✓	39	SE	✓
10	NE	✓	40	NE	✓
11	NE	✓	41	NW	✓
12	SE	✓	42	SW	✓
13	SW	✓	43	NE	✓
14	SW	X	44	NE	✓
15	NE	✓	45	NW	X
16	SE	✓	46	SE	
17	NW	✓	47	SE	
18	NW	✓	48	NW	
19	NW	X	49	SW	
20	SE	✓	50	NW	
21	SE	✓	51	NE	
22	NE	✓	52	SE	
23	NW	✓	53	NE	
24	SW	✓	54	NW	
25	NE	✓	55	SW	
26	SW	X	56	SE	
27	SW	✓	57	NW	
28	SE	✓	58	SW	
29	NW	X	59	NE	
30	NE	X	60	SW	

$\frac{10}{45} = 22\%$

Supplementary Figure 1. Behavioral training and testing. A. Performance of a rat in the cue task before the lesion. Both start and goal locations were varied on a pseudorandom schedule, so that no more than 3 consecutive trials involved the same type of journey (e.g., trials 8-10). The cue was placed at the end of the goal arm and the rat had to walk towards it from whichever start arm it was placed in. This procedure rendered spatial location irrelevant. B. Performance of the same rat in the spatial task during the same day as in (A). In this case, the food was initially placed at the end of the West goal arms and the rat had to walk to that location from either the North or the South start arms. When he reached the criterion of 9 correct runs in a succession of 10 trials, the location of the food was changed to the end of the East goal arm, and the same process repeated. The location of the food was changed 3 times so that the animal could not develop a habit type of response to any of the individual allocentric cues around the maze. Trials 1-10, 11-20, 21-30 and 31-40 constitute each a *block* of trials. C. Performance of the same animal, now with a CA3 lesion, in the spatial task during the first day of post-op testing. Note that blocks 2 and 3 reach the maximum allowed of 15 trials and the location of the food is switched even as the rat has not reached criterion. These blocks are referred to as *long blocks*. D. The performance of the same lesioned animal during the same day in the cue task.

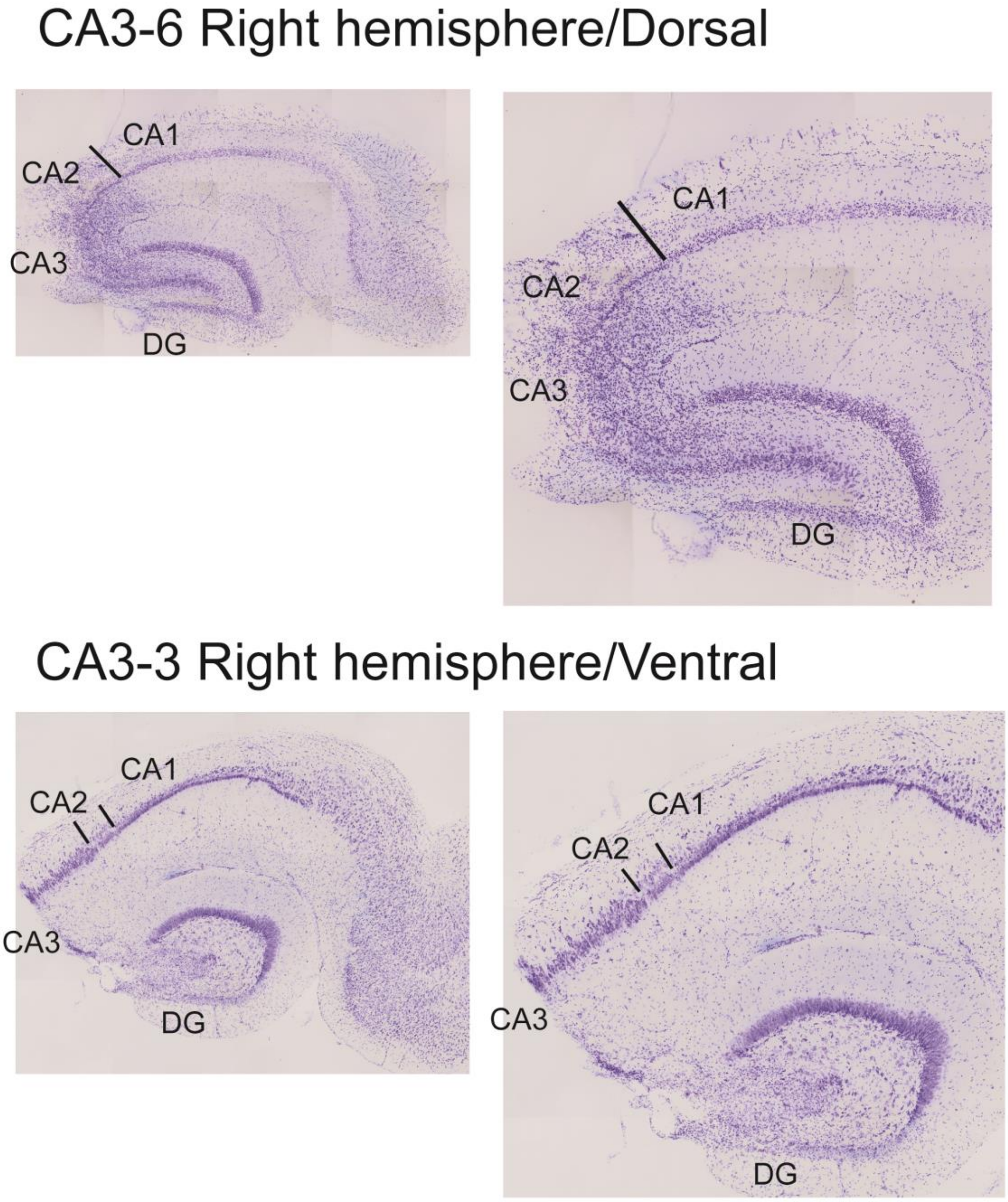

Supplementary Fig. 2. Sections from hippocampal slices obtained from CA3 lesioned rats at the end of electrophysiological recordings. CA3 is largely lesioned along the dorsal-ventral axis, although the lower blade of DG has some damage (asterisk) as well. Lines indicate the border between CA2 and CA3 (where lesion did not extend into CA2); and CA2 and CA1. Images are not to scale with respect to one another.

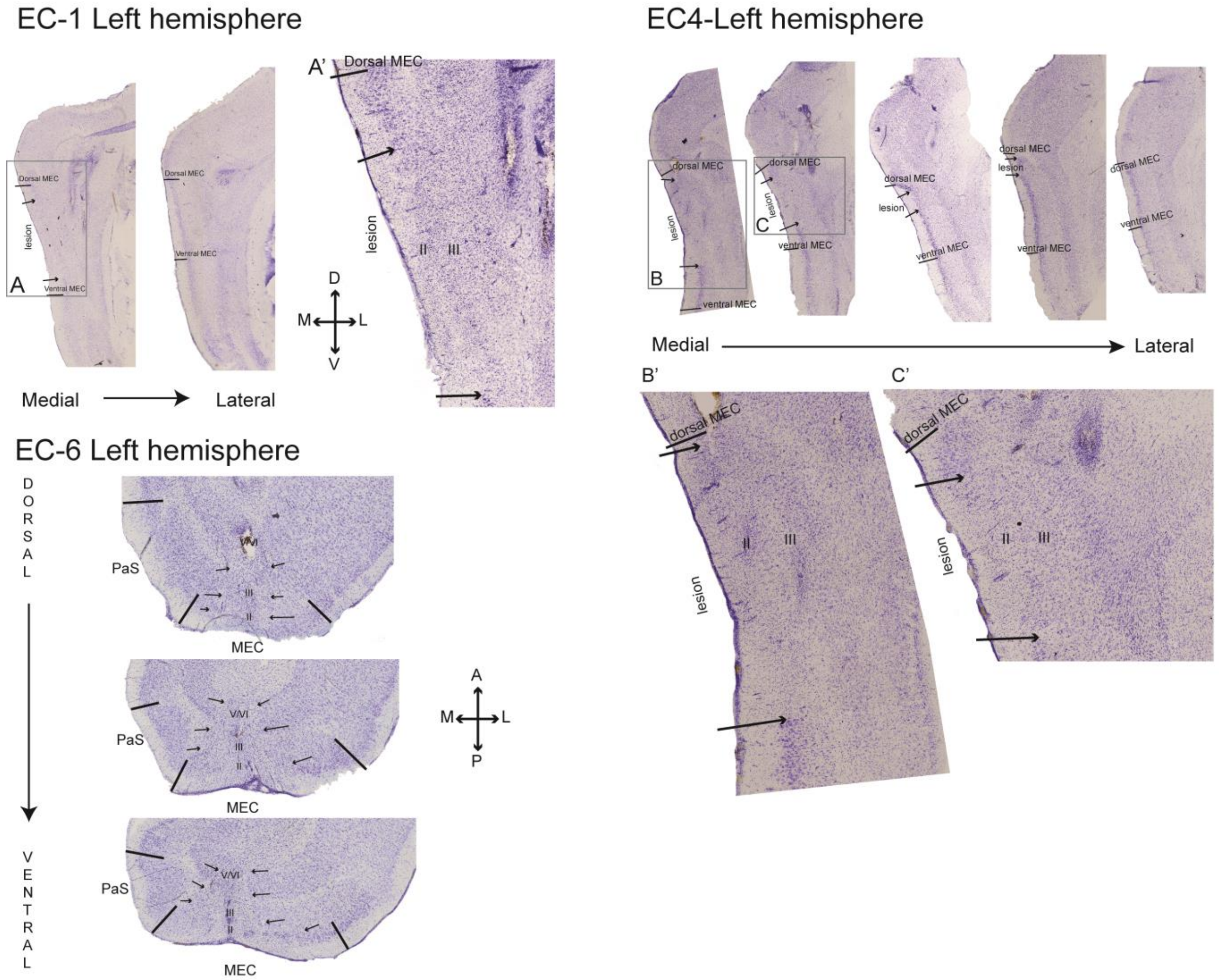

Supplementary Fig. 3. Sections through EC from MEC lesioned rats. In EC-4, the most lateral section in the upper row does not encompass MEC. Areas within the gray rectangles, which center on the lesions, are shown enlarged. To gain a more complete assessment of the damage, some tissue was sectioned sagitally (e.g., EC-1, EC-4) and some horizontally (e.g., EC-6). Damage at the cannula insertion point was mechanical and the neurotoxin did not spread in adjacent LEC, or presubiculum areas. Neurotoxin damage to parasubiculum (PaS) was minimal. It should be noted in the horizontal example, EC-6, that the ventral section is located in the intermediate MEC. Lines represent the MEC borders, arrows represent the limits of the lesioned area, and Roman numerals represent the layers of the MEC. Images are not to scale with respect to one another.
