# Supplementary material for: Relative contributions of CA3 and medial entorhinal cortex to memory in rats
Source: Front Behav Neurosci. 2014 Aug 28;8:292. doi: 10.3389/fnbeh.2014.00292 (PMC4148030; doi:10.3389/fnbeh.2014.00292)
Supplement: Supplementary file 2 [file DataSheet1.DOCX]

|  | | **Food restriction & handling** | **Pre-op training** | | **Surgery** | **Post-op training** | | **Physiology** | **Histology** | **Included in final analysis** |  |
| --- | --- | --- | --- | --- | --- | --- | --- | --- | --- | --- | --- |
|  |  |  | cue | spatial |  | cue | spatial |  |  |  |  |
| **Set1** | **Ctr1** | ✓ | - | - | - | - | - | ✓ | - | ✓ |  |
|  | **Ctr2** | ✓ | - | - | - | - | - | ✓ | - | ✓ |  |
|  | **Ctr3** | ✓ | ✓ | - | - | - | - | ✓ | - | ✓ |  |
|  | **Ctr4** | ✓ | ✓ | - | - | - | - | ✓ | - | ✓ |  |
|  | **Ctr5** | ✓ | - | ✓ | - | - | - | ✓ | - | ✓ |  |
|  | **Ctr6** | ✓ | - | ✓ | - | - | - | ✓ | - | ✓ |  |
|  | | | | | | | | | | | |
| **Set2** | **CA3-2** | ✓ | ✓ | ✓ | CA3 lesion | days1-5 | days1-5 | ✓ | ✓ | ✓ |  |
|  | **CA3-3** | ✓ | ✓ | ✓ | CA3 lesion | days1-5 | days1-5 | ✓ | ✓ | ✓ |  |
|  | **CA3-5** | ✓ | ✓ | ✓ | CA3 lesion | days1-5 | days1-5 | ✓ | ✓ | ✓ |  |
|  | **CA3-6** | ✓ | ✓ | ✓ | CA3 lesion | days1-5 | days1-5 | ✓ | ✓ | ✓ |  |
|  | **CA3-7** | ✓ | ✓ | ✓ | CA3 lesion | days1-5 | days1-5 | - | ✓  Fig 2A | ✓ |  |
|  |  | | | | | | | | | | |
|  | **EC-1** | ✓ | ✓ | ✓ | MEC lesion | days1-2 | days1-2 | ✓ | ✓ | ✓ |  |
|  | **EC-2** | ✓ | ✓ | ✓ | MEC lesion | days1-2 | days1-2 | ✓ | ✓ | ✓ |  |
|  | **EC-3** | ✓ | ✓ | ✓ | MEC lesion | days1-2 | days1-2 | ✓ | ✓ | ✓ |  |
|  | **EC-4** | ✓ | ✓ | ✓ | MEC lesion | days1-2 | days1-2 | ✓ | ✓ | ✓ |  |
|  | **EC-6** | ✓ | ✓ | ✓ | MEC lesion | days1-5 | days1-5 | ✓ | ✓ | ✓ |  |
|  | **EC-8** | ✓ | ✓ | ✓ | MEC lesion | days1-5 | days1-5 | ✓ | ✓ | - |  |
|  | **EC-9** | ✓ | ✓ | ✓ | MEC lesion | days1-5 | days1-5 | ✓  recording with EC in slice | - | ✓ |  |
|  | **EC-10** | ✓ | ✓ | ✓ | MEC lesion | days1-5 | days1-5 | - | ✓ | - |  |
|  |  | | | | | | | | | | |
|  | **TC-1** | ✓ | ✓ | ✓ | sham | days1-5 | days1-5 | ✓ | ✓ | ✓ |  |
|  | **TC-2** | ✓ | ✓ | ✓ | sham | days1-5 | days1-5 | ✓ | ✓ | ✓ |  |
|  | **TC-3** | ✓ | ✓ | ✓ | sham | days1-5 | days1-5 | ✓ | ✓ | ✓ |  |
|  | **TC-4** | ✓ | ✓ | ✓ | sham | days1-5 | days1-5 | ✓ | ✓ | ✓ |  |
|  |  |  |  |  |  |  |  |  |  |  |  |
| **FG** | **FG1** | - | - | - | FG injection | - | - | - | ✓ | ✓ |  |
|  | **FG2** | - | - | - | FG injection | - | - | - | ✓ | ✓ |  |
|  | **FG3** | - | - | - | FG injection | - | - | - | ✓ | ✓ |  |
|  | **FG4** | - | - | - | FG injection | - | - | - | ✓ | ✓ |  |
|  | **FG10** | - | - | - | MEC lesion/FG injection | - | - | - | ✓ | ✓ |  |
|  | **FG11** | - | - | - | MEC lesion/FG injection | - | - | - | ✓ | ✓ |  |

**Supplementary Table 1**: Behavioral training, surgery, physiology and histology procedures. The last column indicates all animals whose data have contributed to the conclusions of the paper.
